# Rapid Changes in Gene Expression Dynamics in Response to Superoxide Reveal SoxRS-Dependent and Independent Transcriptional Networks

**DOI:** 10.1371/journal.pone.0001186

**Published:** 2007-11-14

**Authors:** Jeffrey L. Blanchard, Wei-Yun Wholey, Erin M. Conlon, Pablo J. Pomposiello

**Affiliations:** 1 Department of Microbiology, University of Massachusetts, Amherst, Massachusetts, United States of America; 2 Department of Mathematics and Statistics, University of Massachusetts, Amherst, Massachusetts, United States of America; Tufts University, United States of America

## Abstract

**Background:**

SoxR and SoxS constitute an intracellular signal response system that rapidly detects changes in superoxide levels and modulates gene expression in *E. coli*. A time series microarray design was used to identify co-regulated SoxRS-dependent and independent genes modulated by superoxide minutes after exposure to stress.

**Methodology/Principal Findings:**

*soxS* mRNA levels surged to near maximal levels within the first few minutes of exposure to paraquat, a superoxide-producing compound, followed by a rise in mRNA levels of known SoxS-regulated genes. Based on a new method for determining the biological significance of clustering results, a total of 138 genic regions, including several transcription factors and putative sRNAs were identified as being regulated through the SoxRS signaling pathway within 10 minutes of paraquat treatment. A statistically significant two-block SoxS motif was identified through analysis of the SoxS-regulated genes. The SoxRS-independent response included members of the OxyR, CysB, IscR, BirA and Fur regulons. Finally, the relative sensitivity to superoxide was measured in 94 strains carrying deletions in individual, superoxide-regulated genes.

**Conclusions/Significance:**

By integrating our microarray time series results with other microarray data, *E. coli* databases and the primary literature, we propose a model of the primary transcriptional response containing 226 protein-coding and sRNA sequences. From the SoxS dependent network the first statistically significant SoxS-related motif was identified.

## Introduction

Aerobic metabolism produces reactive oxygen species that expose cells to a chronic risk of oxidative damage. In addition to this chronic exposure, bacterial cells that engage the immune system of mammals can be exposed to acute level of oxidants, produced by specialized cells as strong bacteriocidal agents. In response to chronic and acute oxidative stress, bacteria have evolved signal transduction pathways that sense changes in oxidant levels and modulate gene expression before extensive damage is realized. The best-characterized sensor-regulator systems that respond to oxidative stress are the OxyR and SoxRS systems in *E. coli,* which regulate the responses to hydrogen peroxide and superoxide, respectively [1], [2].

OxyR is a member of the LysR protein family that both senses intracellular hydrogen peroxide levels, and binds to DNA at promoter regions where it regulates transcriptional initiation. The DNA binding capacity of OxyR is modulated by the reversible oxidation of the free thiols in two cysteines (C199 and C208) to a disulfide bond [3]. Only oxidized OxyR activates transcription of target genes, which collectively contribute to prevent and alleviate oxidative damage. OxyR activates the expression, among others, of *katG* (catalase), *dps* (a DNA binding protein), *gor* (glutathione reductase) and *grxA* (glutaredoxin 1). Since OxyR is reduced by glutaredoxin 1, the response is auto-regulated [3].

**Figure 1:**
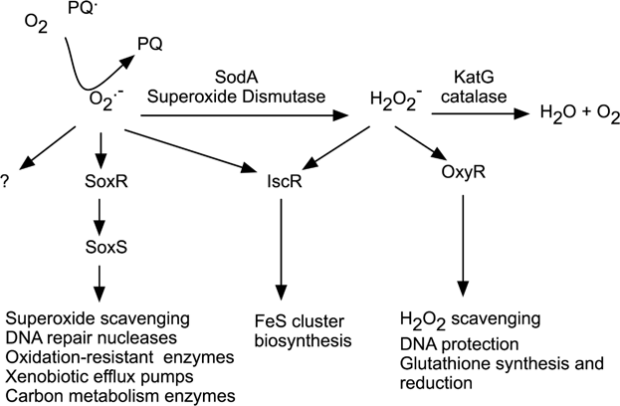
The paraquat-induced transcriptional and metabolic cascade. Paraquat generates superoxide inside the cell by NADPH-mediated reduction of oxygen. Superoxide is sensed by SoxR, which enhances the expression of the transcription factor SoxS, which in turn stimulates other downstream targets. One of the SoxS-regulated genes, *sodA* (superoxide dismutase), converts superoxide into hydrogen peroxide, which acts as a signal to enhance transcription of genes through OxyR. *katG* (catalase), a hydrogen peroxide scavenger is an OxyR-regulated gene. Superoxide and hydrogen peroxide also directly affect IscR modulation of a set of genes involved in iron sulfur cluster biogenesis.

A simplified model of the superoxide response in *E. coli* is shown in Fig. 1. The response to superoxide is partially built around the reversible oxidation of a sensor, SoxR, which enhances the expression of a regulator, SoxS. Electronic paramagnetic resonance and chemical analysis revealed that SoxR is a dimer, containing a [2Fe-2S] cluster per monomer [4]. The reversible oxidation of the [2Fe-2S]^+1^ clusters to [2Fe-2S]^+2^ is sufficient for the activation of SoxR [5], [6]. In this oxidized, activated form, SoxR induces the transcriptional initiation of *soxS*, coding for the AraC-family protein SoxS. SoxS binds to the promoter at target genes, seemingly regulated only by its intracellular concentration. However, SoxS is not only regulated at the transcriptional level by SoxR, but also proteolytically by the Lon protease [7]–[9]. SoxS modulates the expression of genes that code for superoxide dismutase, oxidation-resistant biosynthetic enzymes, the DNA repair nuclease Endonuclease IV, xenobiotic efflux pumps and carbon metabolism enzymes [2], [10].

Three other *E. coli* transcriptions factors, MarA, Rob and YkgA, share considerable sequence homology with SoxS [11] due to recent gene multiplication events in enteric bacteria (Blanchard *et al.*, unpublished data). SoxS positively regulates *marA,* and negatively regulates *rob*, although Rob, MarA and SoxS regulate the expression of a similar set of genes [2], [12]. However, the transcription factors differentially modulate particular promoters, with the consequence that over-expression of SoxS leads to greater superoxide resistance than over expression of MarA [13]. The function or targets of YkgA have not been identified.

Protein radiolabeling and 2D-gel analysis revealed that the expression of at least 80 polypeptides is activated by oxidative stress [14], but many of these proteins were not identified. To identify the genes responsive to superoxide and under the control of the SoxS protein, expression arrays were used to measure differences in transcript levels 45 minutes after exposure to paraquat, a generator of superoxide, and SoxS over expression [15]. Out of 16 known superoxide-regulated genes at the time, 14 significantly increased in the gene array experiments and over a hundred other genes were differentially expressed. Several biological processes in the superoxide response were implicated by these experiments including pathways that reconstitute NADH and NADPH pools, iron transport and storage, sugar and amino acid transport, detoxification, protein modification, osmotic protection, and peptidoglycan synthesis. SoxS-regulated genes identified in these microarray experiments and in past genetic screens do not form complete or even partial pathways, but they may represent key metabolic steps that are sensitive to oxidants.

Some of the genes implicated in the oxidative stress response by the expression array studies were insensitive to stimulation by paraquat using reporter genes [12]. Based on these experiments the total number of directly activated promoters in the SoxS/MarA/Rob regulon may be less than 40 [12]. The discrepancy may be a consequence of the use of early filter-based hybridization technology with few replicates or differences in environmental conditions and genetic background between the studies.

Although comprehensive, previous global studies of oxidation-responsive genes have been limited to steady-state measurements sampled at a single time point after long-term exposure to stress. Responses to stress are typically dynamic, self-regulated and often transient, and therefore, the kinetic analysis of gene expression is necessary to characterize transcription under stress. To define the early response to superoxide, and the corresponding role of the SoxRS system, we designed a time series assay to measure genome-wide RNA levels using DNA microarrays. Our experimental approach was to mimic a rapid rise in superoxide levels by the addition of paraquat, a superoxide-producing agent. The RNA levels of isogenic *wild type* and *ΔsoxR E. coli* strains were measured every two minutes after exposure to paraquat to monitor the early transcriptional response. This approach allowed the discrimination of SoxR-dependent and independent effects. The data obtained was combined with a set of prior knowledge from databases, and manually added from the literature to build a model of the superoxide response network that includes 226 primary response genes. The role of SoxR-dependent genes in superoxide defense was then tested using precise deletion strains of genes.

## Results

### Identification of paraquat-induced transcriptional patterns

To determine the immediate transcriptional response of *E. coli* to superoxide, we conducted a time series assay of the genome-wide mRNA levels immediately following the addition of paraquat (PQ), a redox-cycling agent that produces superoxide intracellularly at the expense of NADPH oxidation [16]. The resulting superoxide is detoxified by superoxide dismutase, producing hydrogen peroxide. The *wild type* strain MG1655 was grown in EZ Rich Defined Medium, a modification of Neidhardt's Supplemented MOPS Defined medium [17] that includes amino acids, nucleotides, vitamins and trace elements in known concentration; and glucose as carbon source. After three cell doublings, and when cultures reached an OD_600_ = 0.5, 250 µM paraquat was added. At this concentration paraquat triggers the SoxRS transcriptional cascade, but only has a negligible effect on exponential growth rate in EZ Rich Defined Medium evidenced by both optical density and ribosomal transcript levels (Supplemental Figures S1 and S2). Samples were taken immediately before exposure to paraquat, and every 2 minutes after exposure, for 10 minutes. Total RNA was extracted from culture samples, and the genome-wide mRNA levels were measured using Affymetrix expression microarrays.

**Figure 2:**
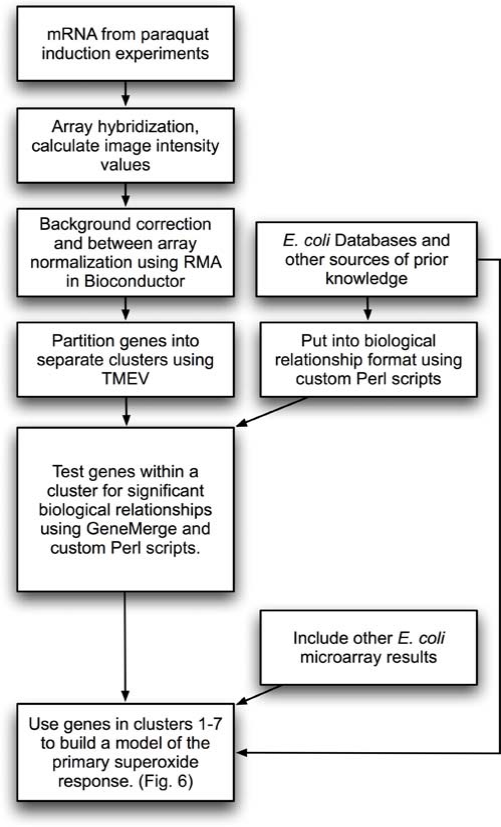
Expression analysis work flow.

Figure 2 contains an outline of our data analysis work flow. The hybridization intensity values from the individual arrays were used to calculate RNA expression levels by correcting for background variation, normalizing across individual arrays and summarizing the individual probe pair data using robust multi-array average (RMA) as implemented in Bioconductor [18]. An initial time course experiment with the *wild type* strain demonstrated that a large and rapid response was induced by paraquat. This time series experiment was repeated with the *wild type* and a *soxR* deletion strain, resulting in 18 microarray assays from the three time course experiments (two sets for *wild type* and one set for *ΔsoxR*).

To assess the quality of the microarray data we graphically examined the images and the distribution of probe intensities for each array and used the probe-level modeling procedures provided by affyPLM [19] in Bioconductor including the images of the weights and residuals, normalized unscaled errors and the relative log expression method. No significant differences between the temporal patterns for the two *wild type* replicates were detected.

To identify putative regulatory networks within the antioxidant response, clustering methods were used to partition the data according to RNA expression patterns through time. The prominent paraquat induced genes could easily be identified in the clustering results. However, there is no generally accepted procedure for determining the biologically relevant number of clusters and cluster analysis is not able to confirm the validity of these groupings. To overcome these problems we developed an approach called “Biological Relationship Analysis”.

### Biological Relationship Analysis can be used to validate clustering results

Biological Relationship Analysis consists of four steps: (1) Formatting biological relationship data files derived from *E. coli* research databases and published data sets. (2) Partitioning the microarray data results into groups of genes with similar expression patterns using clustering methods. (3) Applying a statistical test to determine whether genes having a relationship are over represented in a group. (4) Repeating steps #2 and #3 to determine the appropriate clustering method and number of clusters.

The biological relationship data sets are derived from the *E. coli* research databases and published data sets including; regulatory interactions [20], gene functional categories [21], [22], operons [23], metabolic interactions [21], protein-protein interactions [24], and protein complex associations [21] by extracting information on relationships between genes using custom Perl programs. The information captured is of the form “gene” “relationship” “gene”. Here are a few examples of the data types: 1) “SoxS” “transcriptionally regulates” “*sodA*”. 2) “*sufS*” “iron sulfur cluster synthesis” “*sufD*”. 3) “*sufS*” “operon” “*sufD*”. 4) “*rpsE*” “tandem affinity purification with” “*rpsF*”. 5) “*rpsE*” “part of ribosome with” “*rpsF*”.

Several standard clustering methods were evaluated using a range of cluster numbers from 25 to 500 including the expression values calculated from all MG1655 specific probe sets. We then tested whether a cluster contained more of a particular biological relationship than would be expected by random chance using the hypergeometric distribution with a modified Bonferroni correction as implemented in GeneMerge [25]. Based on this test, we chose to present the K-means clustering results using Euclidean distance metrics and a relatively small number (50) of clusters. This method grouped together the previously identified SoxS-regulated genes, and did not include a cluster containing RpoH-regulated genes which are likely to be the result of secondary regulation mediated by SoxS through RpoH (see below).

**Figure 3:**
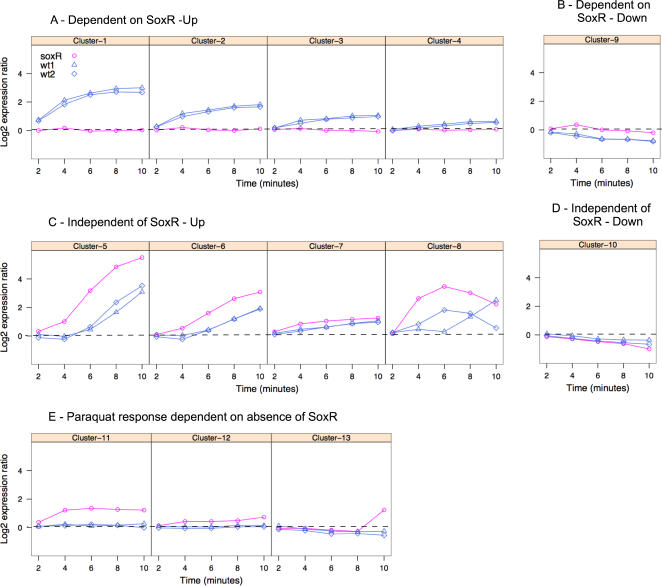
Major paraquat response clusters. Each graph shows average log_2_ expression values as a function of time of all genes in a cluster relative to time zero. The groups are derived from K-means clustering on all three time-series experiments.

Clusters 1, 2 and 3 (Fig. 3) included 22 of the 23 previously described SoxS-regulated genes. The lone exception is *rob*, which is negatively regulated by SoxS. These three clusters include 138 genic regions, including 112 putative protein-coding genes, 3 sRNAs and 23 other intergenic regions. The genes in each cluster shown in Figures 3 are listed in Supplemental Table S1. Many SoxS targeted genes are also regulated by MarA and Rob and thus MarA and Rob appeared as significant transcription factors for these groups. In our biological relationship analysis, the only common biological attributes for genes in cluster 1, 2 and 3, other than “regulated by SoxS, MarA and Rob”, were that some genes are on the same operon and form transporter complexes (see Supplemental Table 2).

The clustering methods also distinguished SoxR-independent Fur and CysB-regulated groups of genes and a cluster containing OxyR, IscR and BirA-regulated genes (Fig. 3 - clusters 5, 6, 7 and 8). These transcription factors govern iron metabolism (Fur), iron sulfur cluster synthesis (IscR), sulfur metabolism and cysteine synthesis (CysB), biotin synthesis (BirA) and hydrogen peroxide related metabolism (OxyR). Paraquat treatment resulted in a faster rise in expression values in the absence of SoxR in all four of these clusters (Fig. 3).

Other patterns are evident in cluster results (Fig. 3). Cluster 4 is enriched in RpoH-regulated genes involved in protein folding and processing, including *dnaJ, dnaK, hslU, hslV, htpG,* and *htpX*. Interestingly, the paraquat-induced expression of the heat shock RNA polymerase sigma factor, RpoH, is SoxR-dependent. In addition, there are genes with levels that increase in *ΔsoxR* in response to paraquat but not in the *wild type* (clusters 11, 12, 13) and genes with levels that decrease in response to paraquat (cluster 9 and 10). Clusters 11 and 12 contain some TCA cycle genes (succinate dehydrogenase, 2-oxoglutarate dehydrogenase and succinyl-CoA synthetase) that are regulated by ArcA and Fnr, and additional genes involved in iron metabolism and cysteine synthesis. Twelve of the 17 genes in cluster 13 function in purine metabolism and are regulated by PurR.

**Figure 4:**
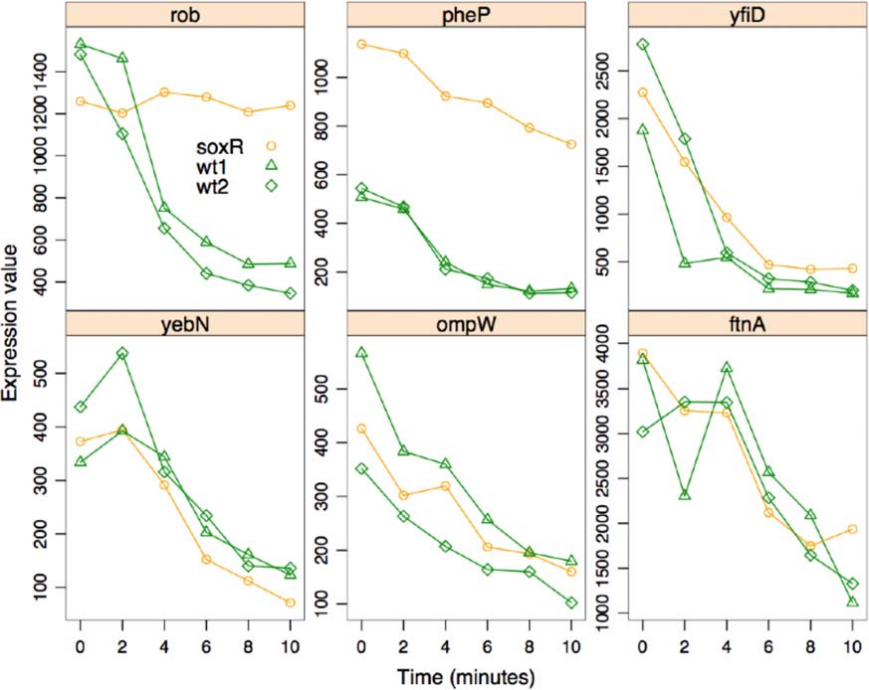
Down-regulated Genes. Each graph shows changes in the expression values of each individual gene in cluster 14.

Only one cluster, containing six protein-coding genes, contained genes whose expression levels decreased with a similar amplitude (Fig. 4) as the genes represented by the mean cluster values shown in Figure 3. This cluster consists of genes having SoxR-dependent and independent transcriptional modulation, because the overall pattern is fairly distinct with respect to the other clusters.

Overall, 32 of the 50 clusters had biological relationships with an e-value less than 0.01 and over 231 terms were associated with those 32 clusters (Supplemental Table S2). Many of the clusters contained genes that were marginally affected by paraquat treatment, but still had significant biological relationships. Further inspection revealed that many of the genes with statistically significant biological relationships in these clusters had small changes that nonetheless covaried over the three time series experiments. These results suggest that kinetic clustering can reveal common biological function, even if the induction ratios involved are relatively small.

### An integrated regulatory model of the superoxide response

**Figure 5:**
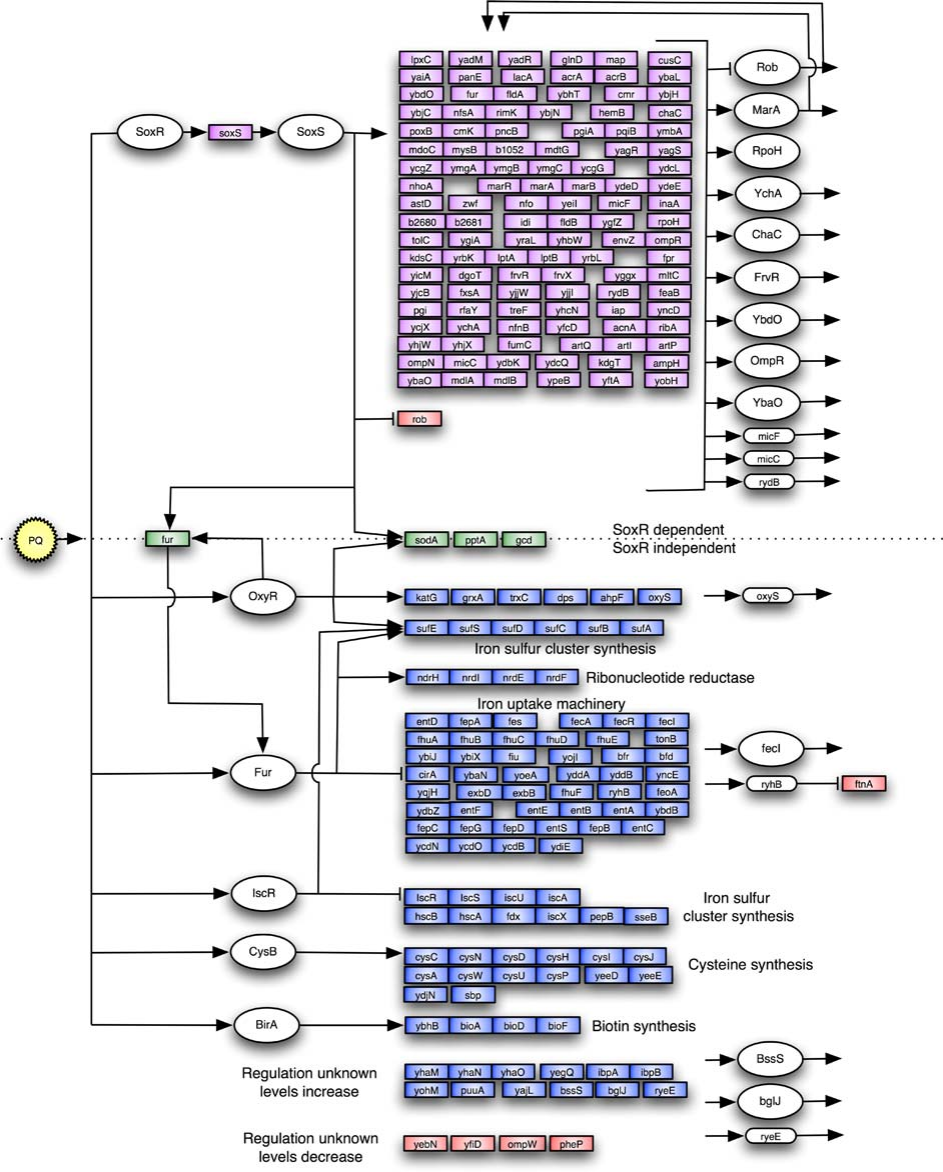
A model of the superoxide transcriptional response. This model summarizes the transcriptional response to paraquat, and consists of protein-coding genes and sRNAs present in the clusters 1–3 and 5–8 shown in Figure 3, along with their regulators and putative targets. It is a synthesis of our microarray results, interactions in RegulonDB, genome annotation and the primary literature related to the regulation of these primary paraquat responsive genes. Thus, it does not show other genes regulated by these transcription factors, nor does it include all regulatory factors that may govern these genes. Regulatory proteins are represented by ovals, sRNAs are represented by rounded rectangles and mRNAs are represented by boxes. SoxR-dependent transcripts that increase are shaded magenta, SoxR-independent transcripts are shaded blue, transcripts that are SoxR-dependent, but still have some residual paraquat response are shaded green, transcripts that decrease are shaded red. Regulatory proteins and sRNAs that appear on the right side of the figure are derived from transcripts present in the boxes. Boxes that are directly attached to each other represent chromosomally adjacent genes. Many of the transcripts are also connected as part of common protein complexes, metabolic pathways and in other biological processes. However, this level of biological detail could not be represented clearly in this model.

To capture the major effects of the paraquat response, the genes in clusters 1–3 and 4–7 were evaluated using the biological relationship data, other published microarray data relevant to these genes, and the primary literature. The results were summarized in a conceptual model of the superoxide transcriptional response (Fig. 5). This model involves 226 protein-coding sequences and sRNAs representing approximately 4% of the currently recognized gene products from the *E. coli* MG1655 genome. We will refer to the genes in this model as primary paraquat responsive genes. Clusters that contained genes with smaller or delayed paraquat responses (clusters 4 and 9–13 in Fig. 3), and intergenic regions that have not been previously identified as sRNAs were excluded from the model.

### The SoxRS regulon

In the proposed transcriptional model, SoxS modulates 119 genes (Fig. 5), including all 23 genes annotated as SoxS targets in RegulonDB, and 9 other SoxS-regulated genes described in the primary literature [10]. The remarkably similar kinetic pattern of induction and the high statistical support in the biological relationship analysis of the SoxRS-dependent clusters (Fig. 3) indicates that most genes in these groups that have not previously been shown to be regulated by SoxS are likely to be regulated directly by SoxS.

**Figure 6:**
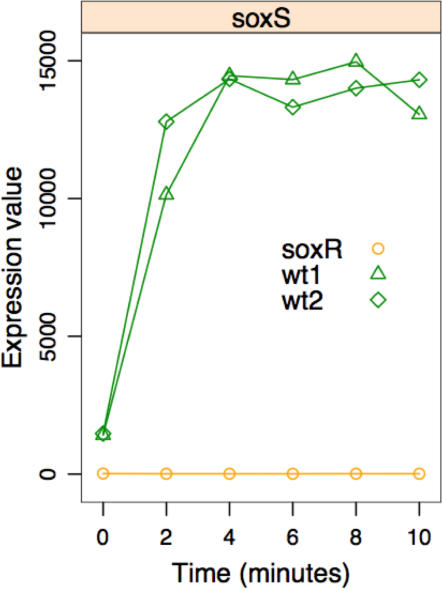
Expression levels of *soxS* mRNA. soxS mRNA levels as are shown as a function of time in the *wild type* and the *ΔsoxR* strain.

In the *ΔsoxR* strain, *soxS* signal levels were below detection level. In the *wild type* strain, before the addition of paraquat, *soxS* expression was detectable (Fig. 6). Following treatment with paraquat, *soxS* transcript levels in the *wild type* strain increased 10-fold relative to the uninduced state to an expression value in the top 99 percentile of all genes. In both time series experiments *soxS* levels were near maximum within 4 minutes in the *wild type* strain. The expression level of SoxS-regulated genes (clusters 1, 2 and 3 in Fig. 3A) in the absence of paraquat is predominately lower in *ΔsoxR* than the *wild type* strain (Data not shown). This observation confirms that SoxS contributes to the expression of the SoxRS regulon during aerobic growth in the absence of exogenous oxidants.

### Phenotypic analysis of strains containing deletions in SoxRS-regulated genes

Changes in superoxide concentration may trigger pathways involved in host-pathogen interactions, since oxygen radicals are frequently used as a killing mechanism by mammalian immune systems, plant defense responses and by other bacteria. Since over half of SoxRS dependent genes identified in the three main clusters have no known function, it is possible that the SoxRS system is part of a larger xenobiotic response system, and regulates many genes that are not necessary for repairing or mitigating oxidative damage. These genes might be required to deal with other compounds derived from the host or to activate genes that help *E. coli* evade a host response, and thus have no role during growth in standard laboratory conditions.

**Figure 7:**
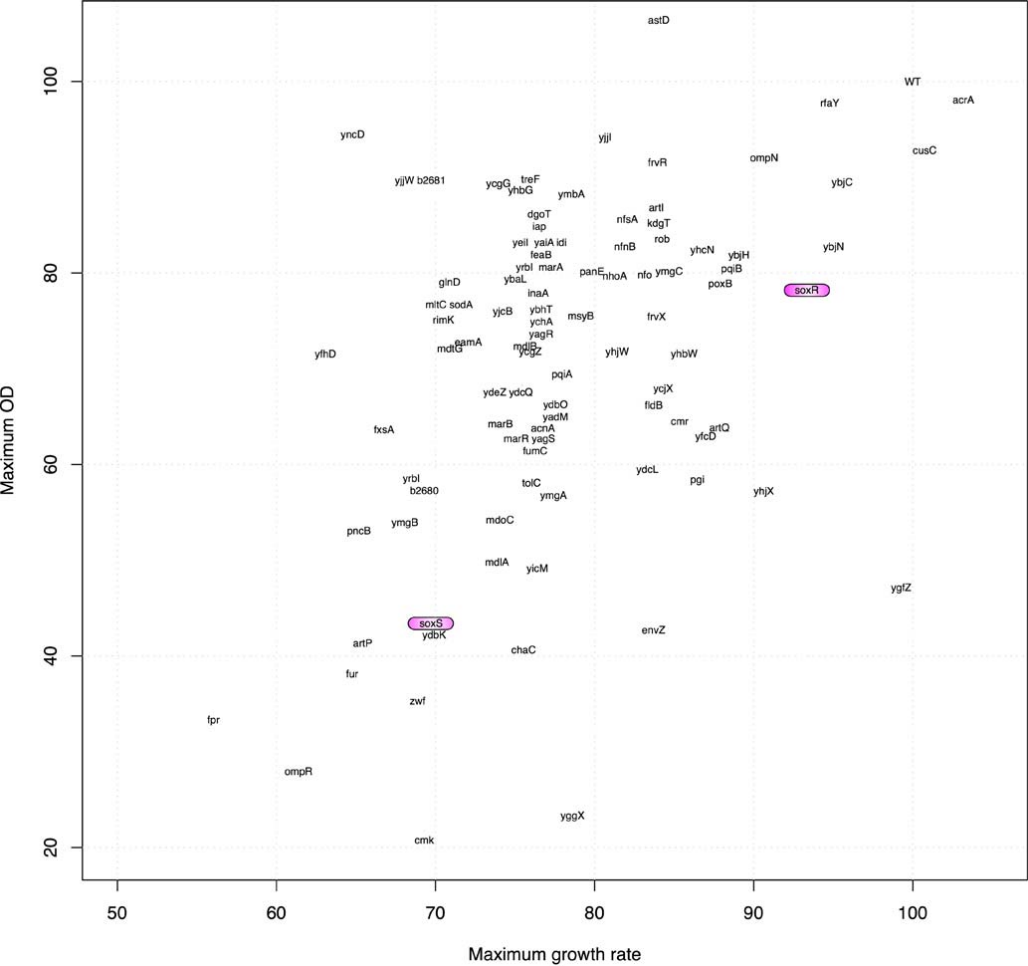
Effect of paraquat on strains carrying deletions in *SoxR*-modulated genes relative to the *wild type*. The values represent the percent difference in final optical density and exponential growth rate in 94 precise deletion strains relative to the *wild type* in presence of paraquat normalized by the change in the deletion strains relative to the *wild type* in absence of paraquat. Therefore, a strain having *wild type* final optical density levels and exponential growth levels in the presence and absence of paraquat would have a value of 100 on both the x and y-axis. A minimum of six replicate growth curves were run for each deletion strain. The results for the *ΔsoxR and ΔsoxS* strains are circled. The following strains were not included because the corresponding genes are essential or are not part of the Keio collection: b1052, *fldA, hemB, ligA, lpxC, map, mdl, ribA, rpoH, yadR, ygiA, yhbN, yraL*, and *yrbK*.

To test this hypothesis, we measured the effect of paraquat on growth rate and optical density at saturation in 94 strains from the KEIO collection [26] carrying precise deletions of individual SoxRS-dependent genes present in clusters 1, 2 and 3 (Fig. 3A). Most gene deletions resulted in a negligible effect on growth in the absence of paraquat, but resulted in a significant deficit when grown in the presence of paraquat (Fig. 7). Nearly all of the mutants had a decreased exponential growth rate and final optical density in the presence of paraquat. A t-test indicated that most gene deletion strains had significantly lower growth rates than the control strain in the presence of paraquat. Interestingly, under these conditions, *ΔsoxS* and *ΔsoxR* strains had significantly different exponential growth rates and final densities. This was unexpected, since *soxS* is the only known regulatory target of SoxR. We repeated the experiments with two independently derived deletion strains from the KEIO collection, and still observed similar results. When we compared the SoxRS data set to a control set of 40 strains with deletions of genes whose mRNA levels are not paraquat-responsive, we found many strains that grew slower in the presence of paraquat (data not shown). Thus, although regulation of a gene by the SoxRS system was a strong predictor of antioxidant function, the sensitivity to superoxide is not exclusive to strains with deletions in SoxRS-regulated genes.

Interestingly, thirty-five SoxRS-regulated *E. coli* genes match genes in the human proteome with an e-value smaller than 10^−4^ (Supplemental Table S3). Not surprisingly, this list of human genes includes important defenses against superoxide like SodA, and many genes involved in NADPH regeneration. These results suggest that metabolic genes conserved from *E. coli* to humans are likely to play a role in mitigating the effects of superoxide damage.

### Motif discovery in gene expression clusters

**Figure 8:**
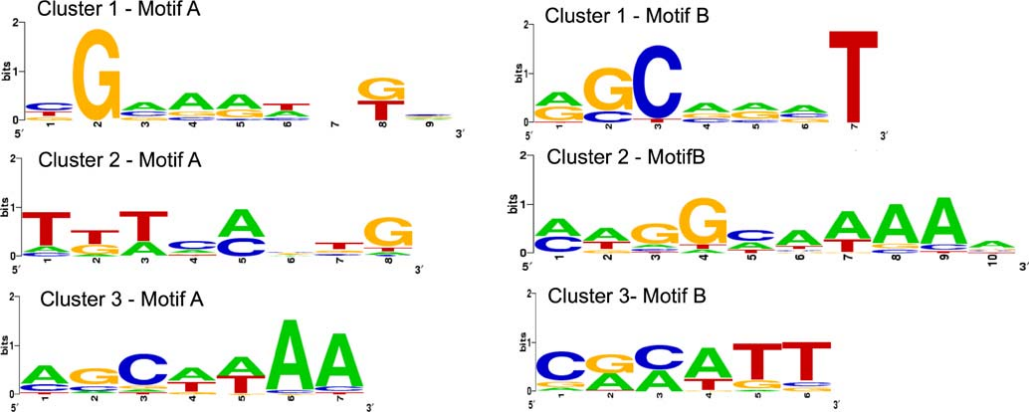
Significant two-block motifs in SoxR-dependent clusters. The two block algorithm implemented in Bioprospector finds a motif with two blocks separated by a variable length gap. The median gap for cluster 1 is 7 with a range of 5 to 9. The median gap for cluster 2 is 6 with a range of 4 to 8. The median gap for cluster 3 is 6 with a range of 5 to 8.

The large number of SoxRS-dependent genes discovered provided an opportunity to identify SoxS regulatory motifs and to identify regulatory motifs in other clusters. We used the motif-finding algorithms implemented in BioProspector [27] to identify motifs in the regulatory sequences of the protein coding genes in the 50 clusters that had an upstream non-coding length of greater than 50 bases (Fig. 8). Of the 50 clusters, we found motifs in 22 clusters that had final scores greater than null scores (data not shown). The motifs derived from upstream regions in clusters 5 and 6 (Fig. 3C) share a common sequence that is identical to the FUR box motif [28]. The consensus sequence in cluster 7 (Fig. 3C) contains a palindrome (ACGCCTGA TCAGGCGT) and most of the other motifs detected also contained palindromes in the consensus sequence.

No significant motifs were detected in the SoxR-dependent clusters 1, 2 and 3 (Fig. 3A) individually, or when they were analyzed as a group. We then tried using a two-block approach to identify motifs that are spanned by a gap that could vary in length between 6 and 30 nucleotides. This algorithm detected significant motifs in all three clusters (Fig. 8). The three motifs share a common GCAAA consensus sequence that is similar to recognition element 2 (YAAA) of the soxbox consensus sequence AYNGCACNNWNNRYYAAAYN. Block A of clusters 1 and 2 (Fig. 3A) has the pattern ANNG, present at the 5′ end of the soxbox. The median spacing between the ANNG and GCAAA sequences in clusters 1 and 2 is one base longer than the spacing between these regions in the soxbox. These results provide further evidence that genes in clusters 1, 2 and 3 are directly regulated by SoxS.

## Discussion

### Biological roles of SoxRS regulon members

A complete list of the SoxRS-regulated genes and their putative products is shown in Supplemental Table S1. Previous research on the physiological role of SoxS regulatory targets has revealed a diverse set of biological functions, including superoxide scavenging, oxidation-resistant biosynthetic enzymes, DNA repair, xenobiotic efflux pumps and carbon metabolism enzymes [2], [10]. In our biological relationship analyses, the only common biological attributes, other than “regulated by SoxS, MarA and Rob”, were that some genes are on the same operon and/or form putative transporter complexes. Thus, given the paucity of knowledge of the biochemical role of most of these genes, there is still a considerable amount of work necessary to assemble an integrated physiological depiction of the superoxide response. As a starting point, we propose three broad functional categories of SoxS-regulated genes that are not adequately represented by the current biological ontologies: (1) NADPH regeneration, (2) removal of xenobiotics and recycling of damaged macromolecules and (3) damage prevention.

Some of the genes regulated by SoxS that may contribute to NADPH regeneration include: *treF, zwf, pgi, gcd, poxB, ydbK, fldA, fldB, fpr, astD, feaB*, and *fumC*. Several of these genes including *zwf, pgi, poxB, acnA, fldA, fldB,* and *fumC* have previously been shown to be under SoxS control (see references in [10]). Upon exposure to hydrogen peroxide, the NADH pool is depleted, and NADPH, which is less reactive with Fe^3+^, functions as the major nicotinamide nucleotide reductant [29]. Thus, just as NAD and NADP have contrasting roles in normal cellular metabolism (degradative vs. synthetic reactions), they also play different roles in the oxidative stress response [29], [30]. Oxidative stress is expected to impose a drain on NADH and NADPH pools because it activates a variety of repair processes that consume reduced pyridine nucleotides, including the OxyR-regulated enzymes glutathione reductase, alkyl hydroperoxide reductase, and thioredoxin reductase. Almost all of these SoxS-regulated genes described above have putative functional homologs in humans based on BlastP searches against the human genome (Supplemental Table S3).

### A significant soxbox-like motif can be detected upstream of SoxS regulated genes using a two-block algorithm with variable length gap

A 20 bp regulatory motif, the *soxbox*, has been proposed based the alignment of sequence regions identified by DNA footprinting, DNA methylation studies and promoter fusion analysis [31], [32]. Genome wide searches of these motifs using information theory based methods identified thousands of possible SoxS binding sites. Several groups have noted that the SoxS motif has low information content (McGuire 2000, Li 2002) and it has not been possible to effectively use the SoxS motifs in cross-species comparisons. Because of these limitations, the current proposed *soxbox* has not been useful in identifying novel genes under the control of SoxS. This inability to predict SoxS binding sites suggests that addition of sequence determinants is necessary for determining SoxS-mediated expression.

Recently, a RNA polymerase pre-recruitment model has been proposed where SoxS acts as a co-sigma factor, binding RNA polymerase in solution before the complex binds to promoters [33]–[35]. Formation of the SoxS-RNA polymerase complex diverts RNA polymerase from UP-element containing promoters to SoxS-dependent promoters [7]. The SoxS-RNA polymerase complex may require sequence specificity in both the RNA polymerase binding sites and the SoxS binding site, resulting in spatially separated sequence motifs for RNAP and SoxS binding [12].

This model motivated us to search the intergenic sequences upstream of genes in the SoxR-dependent clusters 1, 2 and 3 (Fig. 3A) for two motifs separated by varying nucleotide lengths. A statistically significant *soxbox* was not detected using the common one block motif approach. Our inability to detect DNA motifs that match the proposed consensus *soxbox* could interpreted as evidence that clusters 1, 2 and 3 contain genes that are regulated indirectly by SoxS. This is unlikely, since if these genes were regulated by a factor that requires induction by SoxS the activation would be delayed with respect to know SoxS regulated genes.

The use of a variable length gap between blocks allowed us to detect a significant motif that contained one of the recognition elements of the soxbox in all three clusters. Clusters 1 and 2 shared additional elements of the *soxbox* including the presence of the ANNG motif and the similarity in spacing between blocks A and B and the *soxbox*. The motif is found in, or overlaps with *micF* and *zwf* upstream regions identified by DNAse footprinting [36]. These results provide further evidence that genes in clusters 1, 2 and 3 are directly regulated by SoxS.

### SoxS is a global regulator that induces the expression of other regulators

The SoxR-dependent genes include four known transcriptional regulators (Rob, MarA, Fur and OmpR), a sigma factor (RpoH), five genes annotated as putative transcription factors (YhcA, ChaC, FrvR, YbaO and YbdO), and three known regulatory sRNAs (MicF, MicC and RydB). Thus, SoxS is a global regulator that modulates the expression of other regulators. Although Rob and MarA can regulate the same genes as SoxS, it is not at all clear what their roles are in the paraquat response. Potentially, SoxS and MarA form a feed forward circuit in which *marA* expression results in a further increase in target gene levels. MarA may also regulate a set of genes independent of SoxS. The down regulation of *rob* expression may not result in changes in Rob targets within this time series, although Rob could act as a repressor of some SoxS-targeted genes. *marA*, *rob* and *ybdO* were differentially expressed in response to paraquat in the GenoSys macroarrays [15]. *ybaO* can be transcriptionally activated by Rob [37].

In bacteria, small non-coding sRNAs can perform fine-tuning of gene expression, although the transcripts are just beginning to be identified, and their functions are largely unknown [38]–[43]. Within clusters 1–3 and 5–8 there are 49 genic regions that do not code for proteins. Six previously identified sRNAs are part of the paraquat response model, although the function of RyeE and RydB is unknown. Of the ΔSoxR independent sRNAs, OxyS is the most prominent paraquat-responsive sRNA. OxyR regulates oxyS expression in response to hydrogen peroxide and paraquat. OxyS has been shown to block translation of FhlA, a transcriptional activator, and RpoS, an alternative sigma factor [38]. However, the expression levels of these genes do not change in our assay.

We were startled to observe a significant difference in the exponential growth rate and final optical density of *ΔsoxR and ΔsoxS* strains. Our results suggest that a lack of the *soxS* gene results in a larger defect in resistance to superoxide than the lack of a *soxR* gene. This suggests that there might be alternative mechanisms for activating *soxS* expression in the conditions tested (which were different from conditions used for the micoarray experiment).

### The paraquat induced expression of the Fur regulon is independent of SoxRS

Our microarray time series assays revealed that the expression of most iron acquisition genes rises dramatically in response to paraquat independently of the SoxRS pathway. Nearly all of the genes involved in iron transport and storage whose levels increase under low iron or in the absence of Fur [44] also increase in response to paraquat. In addition to the iron transport and storage genes, ribonucleotide reductase (*nrdHIEF*), *ompW* and *ibpAB* chaperone levels were also higher under low iron conditions [44] and in response to paraquat (Fig. 3C).

An apparent paradox in the iron response is that cells that constitutively express iron import genes are hypersensitive to oxidants [45], but we observed that cells under oxidative stress have increased expression levels of iron import genes. The up-regulation of the Fur regulon suggests that *E. coli* growing under superoxide stress is limited for iron. Indeed, preliminary studies showed that adding 2-dipyrydil, an iron chelator, to *E. coli* cultures growing in the presence of PQ reduced growth rate dramatically. The same chelator added in the absence of PQ had no effect on growth rate (Bain and Pomposiello, unpublished).

A direct regulatory connection between oxidative stress and iron metabolism was shown by Zheng *et al*., who demonstrated the transcriptional activation of *fur* by SoxS and OxyR [46]. Additionally, generation of hydrogen peroxide can result in oxidation of the Fur-Fe^2+^ complex thereby inactivating Fur repressor function [47]. An alternative hypothesis for the derepression of the Fur regulon is based on the observation that Fe^3+^ does not seem to function as co-repressor. In this scenario, under oxidative stress the Fe^2+^ associated with Fur is oxidized to Fe^3+^, which leads to the expression of Fur-repressed genes. It is also possible that the response is a transient phenomenon caused by oxidative damage to Fur, or that intracellular iron levels are too low to allow to Fur repressor function. *E. coli* contains a small pool of intracellular free Fe^3+^, and superoxide increases levels of intracellular free iron primarily as the result of damage to iron sulfur clusters in fumarase, aconitase, and 6-phosphogluconate dehydratase, enzymes that have a solvent-accessible iron atom [48]. The metabolic fate of this free iron is uncertain, but our results suggest that it can not mediate the repression of the Fur regulon under oxidative stress.

Cysteine can replace iron in driving the Fenton reaction, and homeostatic control of cysteine levels is important in limiting damage by oxidants [49]. CysB and its positive effector N-acetyl-l-serine (NAS) are required for transcriptional activation of all genes of the cysteine regulon [50]. NAS is formed from O-acetyl-l-serine (OAS) in a spontaneous and irreversible reaction. L-cysteine feedback inhibits the synthesis of OAS (and therefore also of NAS) from l-serine [51]. Therefore, reduction of cysteine might cause a transient rise in NAS levels followed by the induction of the CysB regulon (Fig. 3C – cluster 8).

The *suf* and *isc* Fe-S clusters assembly pathways are both induced by hydrogen peroxide and iron chelators, but induction of the *isc* operon seems to operate through direct oxidation of IscR by hydrogen peroxide or superoxide [52], [53] whereas *suf* gene expression is mediated by OxyR [54]. In our microarray data the mRNA levels of genes in both the *isc* and *suf* clusters increased in response to paraquat. The *suf* genes have a similar expression pattern as the iron acquisition genes and continue to rise at the 10-minute mark. The *isc* cluster mRNA levels increase rapidly and then level off around six minutes. However, only a subset of the IscR-repressed genes identified in *ΔiscR* strains [52] are paraquat responsive. This may be the result of differences in transcriptional repression by the apo-IscR proteins and IscR proteins containing Fe-S clusters.

### Decrease of the NADPH pool is likely to have negligible effects on the short-term transcriptional response

Paraquat can affect bacteria by at least two means; the production of superoxide and the depletion of reducing equivalents. In *E. coli* NADPH:paraquat diaphorase activity can be catalyzed by NADPH:ferredoxin oxidoreductase (Fpr), NADPH:thioredoxin reductase (TrxB), and possibly other oxidoreductases [55]. The response of the SoxR-dependent genes is the result of direct reduction of SoxR by superoxide. Are the SoxR-independent genes the result of a decrease in NADPH levels from the reduction of paraquat? The SoxRS-independent aspect of the transcriptional response model can be explained by the known roles of superoxide in directly regulating OxyR and IscR, the role of superoxide in iron and cysteine reduction and the subsequent generation of hydrogen peroxide. Thus, it appears that little if any change in expression is the result of changes in NADPH levels, or other indirect short-term effects.

### Biological Relationship Analysis identifies statistically significant biological relationships in clustering results

The development of the Biological Relationship Analysis method addresses a common critique of microarray studies that they need to be “validated” by an independent method. This criticism is derived from earlier microarray studies in which there was considerable variation between arrays as the result of the array fabrication processes. Because there are limited methods for determining the significance of clustering results, most researchers further test the biological significance of a small subset of the clustering results through using more traditional gene-by-gene approaches. However, this does not in any way validate the clustering results.

A common statistical method for testing whether a particular group of genes with a shared function occurs more frequently in a cluster than would be expected based on random sampling of the microarray data set is the application of the hypergeometric distribution [25]. Our approach uses this statistical test in an iterative fashion to identify statistically significant biological relationships in the clustering results. Our clustering objective for this analysis was to separate the genes likely to be regulated directly by SoxS and those that might be the result of secondary regulation via a SoxS activated regulator. The groupings derived from K-means clustering using Euclidean distance metrics were used because they captured the previously identified SoxS-regulated genes and did not include a cluster containing RpoH-regulated genes, which are likely to be the result of secondary regulation mediated by SoxS through RpoH. It is possible that some genes in the SoxRS regulon are regulated indirectly and that we may have excluded some SoxS-regulated genes from the model that did not have a large response. It is important to note that this statistical test is applied after the clustering analysis and that the prior knowledge of the superoxide response or any other aspect of *E. coli* biology does not bias the clustering methods.

Although we used clustering methods to define the set of transcripts in Figure 5, creating the model required manual inspection of the individual gene profiles and integrating particular interactions and transcription factors from the *E. coli* databases and the published literature. SoxR, OxyR, BirA and CysB transcript levels do not change, and would not have been included in the transcriptional model without prior knowledge of their regulatory mechanisms and targets. In addition, the levels of *fur* and *iscR* increase, although their well-established biological roles are as repressors and their target genes are increasing rather than decreasing.

We also observed that known members of a regulon did not necessarily group in the same cluster. For example, OxyR regulon members were concentrated in cluster 7, but were also part of clusters 5 and 8. Collecting samples more frequently, over a longer time series and with more biological replicates might generate tighter groupings. However, we do not expect all genes in the same regulon to have identical expression patterns. *sodA*, *fur*, *gcd* and *pptA* increase in response to paraquat in the *wild type* and *ΔsoxR*, but the paraquat response is much lower in the *ΔsoxR* strain. In the clustering results *fur*, *sodA* and *pptA* were part of the SoxR-dependent clusters, while *gcd* groups with the SoxR independent clusters. These observations are consistent with the known regulation of these genes by multiple transcription factors.

Only 16 out of 226 primary paraquat responsive genes in our transcriptional model (Fig. 5) were not assigned to a transcription factor. There maybe missing transcription factors in our model, but it seems more likely that these genes belong to the OxyR, IscR, Fur, or CysB regulons. There are genes that have been previously attributed to the Fur regulon that are missing from the model. Some of these genes do not have a strong response within the 10-minute time series, however they may be a part of the Fur regulon that is not expressed in response to superoxide. These results emphasize the importance of using kinetic approaches to study stress responses.

### Conclusions

*E. coli* responds very rapidly to changes in superoxide levels generating distinct expression patterns within the first 10 minutes. By integrating our microarray time series results with other microarray data, *E. coli* databases and the primary literature, we propose a model of the primary transcriptional response. There is still an enormous amount of work needed to compile an integrated physiological model of the superoxide response, since we do not understand the biochemical roles of most of the SoxS-regulated genes. This will require detailed study of many individual genes.

While we framed our discussion of the SoxS regulon in terms of NADPH regeneration, removal and recycling of damaged macromolecules and damage prevention, numerous biological processes appear to be affected by superoxide. These processes undoubtedly have multiple control mechanisms, however unlike other transcription factors, SoxS appears to modulate individual points in these processes and does not in general up regulate complete pathways. Many of the genes regulated by SoxS have homologs in the human genome. Thus, we anticipate that there will be many antioxidant mechanisms conserved between bacteria and eukaryotic organisms beyond the known examples of catalases and superoxide dismutases.

## Materials and Methods

### Bacterial strains

*E. coli* strain MG1655 was used as *wild type* in all experiments. The *ΔsoxR* strain and all other single-gene deletion mutant strains used in this study were derived from MG1655 and are part of the Keio collection of non-essential gene deletions [26].

### Culture growth and RNA extraction

Overnight cultures were inoculated at 1∶100 into 20 ml of EZ Rich Defined Medium (Teknova, Inc). The EZ medium is a slight modification of Neidhardt's Supplemented MOPS Defined medium [17] that includes amino acids, nucleotides, vitamins and oligoelements at defined concentrations; and glucose (0.1%) as carbon source. Cultures were grown in 125 ml Erlenmeyer flasks at 37°C and 250 rpm in a reciprocating water bath. Paraquat (Sigma) was added to growing cultures at a final concentration of 250 µM. Samples (1.4ml) were taken prior to paraquat addition and every 2 minutes following paraquat treatment for the length of the time course, and flash-frozen by immersion of the tubes in liquid nitrogen. The cells were collected by centrifugation for 1 minute at 13,000 rpm, and the total RNA was isolated using Qiagen RNeasy Mini Kit and treated with RNAse-free DNase I. The RNA concentration was determined by absorbance at 260/280nm using an Eppendorf BioPhotometer.

### Microarray processing and calculation of expression values

The cDNA synthesis, array hybridization and imaging were performed at the Genomic Core Facility at the University of Massachusetts Medical Center. The total RNA from each sample was used as template to synthesize labeled cDNAs using Affymetrix GeneChip DNA Labeling Reagent Kits. The labeled cDNA samples were hybridized with Affymetrix GeneChip *E. coli* genome 2.0 Arrays according to Affymetrix guidelines. The hybridized arrays were scanned with a GeneChip Scanner 3000. The resulting raw spot image data files were processed into pivot, quality report, and normalized probe intensity files using Microarray Suite version 5.0 (MAS 5.0). The quality of the microarray data sets were analyzed using probe-level modeling procedures provided by the affyPLM package [19] in BioConductor [56]. Robust Multi-Array Average (RMA) [57] was used for background correction, normalization and calculation of expression values for all 18 samples from the probe intensity files. The calculated expression values and original probe intensity files have been deposited in NCBI'S GEO microarray database (series accession #GSE6992).

### Clustering and biological significance analysis

K-means clustering was used to detect patterns in mRNA expression using distance metrics implemented in the TIGR Multiexperiment Viewer (MeV) [58]. In order to further examine functions of genes and to identify other biological information associated with genes grouped in the same cluster, data was collected on regulatory interactions [20], gene functional categories [21], [22], [59] chromosomal positions and operons [23], metabolic interactions [21], protein-protein interactions [24], and protein complexes associations [21] from the EcoCyc [21], KEGG (Kanehisa et al., 2006), RegulonDB [20], and ASAP databases as well as large data sets in the primary literature [22]–[24]. These data sets were transformed using custom Perl scripts into a general format that linked genes together by their common associations. Each of the clusters was then tested for statistical over representation of the related genes having a common biological relationship using the hypergeometric distribution with a modified Bonferroni correction as implemented in GeneMerge [25]. Perl programs used for the Biological Relationship Analysis, biological relationship data sets, and the full results are available in our website -http://www.micro.umass.edu/micro/blanchard/biorelate_PloS_2007.html.

### Regulatory motif discovery

Sequences found immediately upstream of all protein coding (CDS) regions and ending either at the next CDS region or 800 bases upstream were collected from the *E. coli* MG1655 genome (GenBank record NC90013.gbk) using a custom Perl program. Regions less than 50 bases in length were filtered out. We then used BioProspector to identify top scoring motifs in each cluster. We optimized each motif using the program BioOptimizer [60]. The output of BioOptimizer is the optimal motif width and best set of aligned segments based on a Bayesian motif scoring function. Sequence logos of the motifs were created with Weblogo [61].

### Paraquat sensitivity assay

Paraquat sensitivity was determined by comparing growth curves between untreated and treated cultures relative to untreated and treated cultures of the *wild type* strain. Overnight cultures were diluted 1∶100 into 200 µl of fresh Luria Broth (LB) in 96-well microplates and 10 µl of paraquat stock solution was added for a final concentration of 250 µM. Cell growth was measured by monitoring optical density at 600nm (OD600) every 15 minutes for 11 hours in 96-well microplates in a BioTek microplate reader at 37°C with continuous shaking in between measurements. A minimum of six replicate growth curves were run for each deletion strain. Maximum exponential growth rates were calculated by performing linear regressions every 3 points and finding the maximum slope using custom Perl programs. A t-test was run in the program R to determine if the deletion strains were significantly different from the wild type.[Table pone-0001186-t001]

**Table 1 pone-0001186-t001:** Partial summary of Biological Relationship Analysis results for transcription factors which regulate genes in the clusters shown in Fig. 3.

Cluster	# of genes in cluster	Transcription factor relationship	e-value	Regulatory role
Cluster-1	20	SoxS	2.4×10^−09^	Superoxide response
		MarA	5.4×10^−08^	Multiple antibiotic resistance
		Rob	2.7×10^−07^	unknown
Cluster-2	47	SoxS	5.6×10^−18^	Superoxide response
		MarA	1.6×10^−07^	Multiple antibiotic resistance
		Rob	1.0×10^−03^	unknown
Cluster-3	74	SoxS	1.1×10^−05^	Superoxide response
		MarA	5.9×10^−05^	Multiple antibiotic resistance
Cluster-4	93	RpoH	6.5×10^−06^	Sigma 32, Stress response
Cluster-5	17	Fur	2.4×10^−22^	Iron transport, enterobactin synthesis
Cluster-6	35	Fur	2.7×10^−25^	Iron transport, enterobactin synthesis
		OxyR	2.7×10^−07^	Hydrogen peroxide response
Cluster-7	57	IscR	1.1×10^−07^	Iron sulfur cluster synthesis
		OxyR	1.4×10^−04^	Hydrogen peroxide response
		BirA	4.7×10^−05^	Biotin synthesis
Cluster-8	17	CysB	1.7×10^−21^	Cysteine synthesis
Cluster-9	46	LeuO	1.8×10^−04^	Leucine synthesis
Cluster-10	73	Fnr	6.3×10^−06^	Global regulator of anaerobic growth
		GatR	5.7×10^−07^	Regulator of galactitol metabolism
Cluster-11	37	LldR	3.1×10^−06^	L-lactate utilization
		PdhR	1.6×10^−04^	Repressor of pyruvate dehydrogenase
Cluster-12	102	ArcA	5.7×10^−04^	Aerobic respiration
Cluster-13	20	PurR	1.0×10^−25^	Purine synthesis

## Supporting Information

Supplemental Figure S1 Growth of strain MG1655 exposed to PQ. A culture of E. coli strain MG1665 (wt) was started by dilution of an overnight culture 1/100 in fresh EZ medium. The culture was grown at 37°C with strong aeration (250 rpm). At time = 0, the culture was split and one half was left untreated, while the other half was exposed to 500 µM paraquat.
(0.70 MB TIF)

Supplemental Figure S2 The expression pattern of a SoxR dependent cluster (cluster 1) and a cluster containing 49 ribosome-related genes (cluster 31). Each graph shows mean change in the average ratio of log2 expression values of all genes in the cluster relative to time zero. The groups are derived from K-means clustering on all three time-series experiments.
(0.70 MB TIF)

Supplemental Table S1 List of genes from the clusters in Figure 3.
(0.68 MB DOC)

Supplemental Table S2 Biological Relationship Analysis results for all 50 clusters.
(0.26 MB DOC)

Supplemental Table S3 Human homologs of SoxS regulated genes identified by BlastP.
(0.06 MB DOC)
