# Correction: Rapid Changes in Gene Expression Dynamics in Response to Superoxide Reveal SoxRS-Dependent and Independent Transcriptional Networks

**DOI:** 10.1371/annotation/5cba04eb-5172-43a7-ad92-10efcd3858c9

**Published:** 2012-11-08

**Authors:** Jeffrey L. Blanchard, Wei-Yun Wholey, Erin M. Conlon, Pablo J. Pomposiello

Supplemental Figure S1 is a duplicate of Figure S2. Please view the correct Figure S1 here: 

[^]

