# Supplementary figures and images for: Rapid Changes in Gene Expression Dynamics in Response to Superoxide Reveal SoxRS-Dependent and Independent Transcriptional Networks
Source: PLoS One. 2007 Nov 14;2(11):e1186. doi: 10.1371/journal.pone.0001186 (PMC2064960; doi:10.1371/journal.pone.0001186)

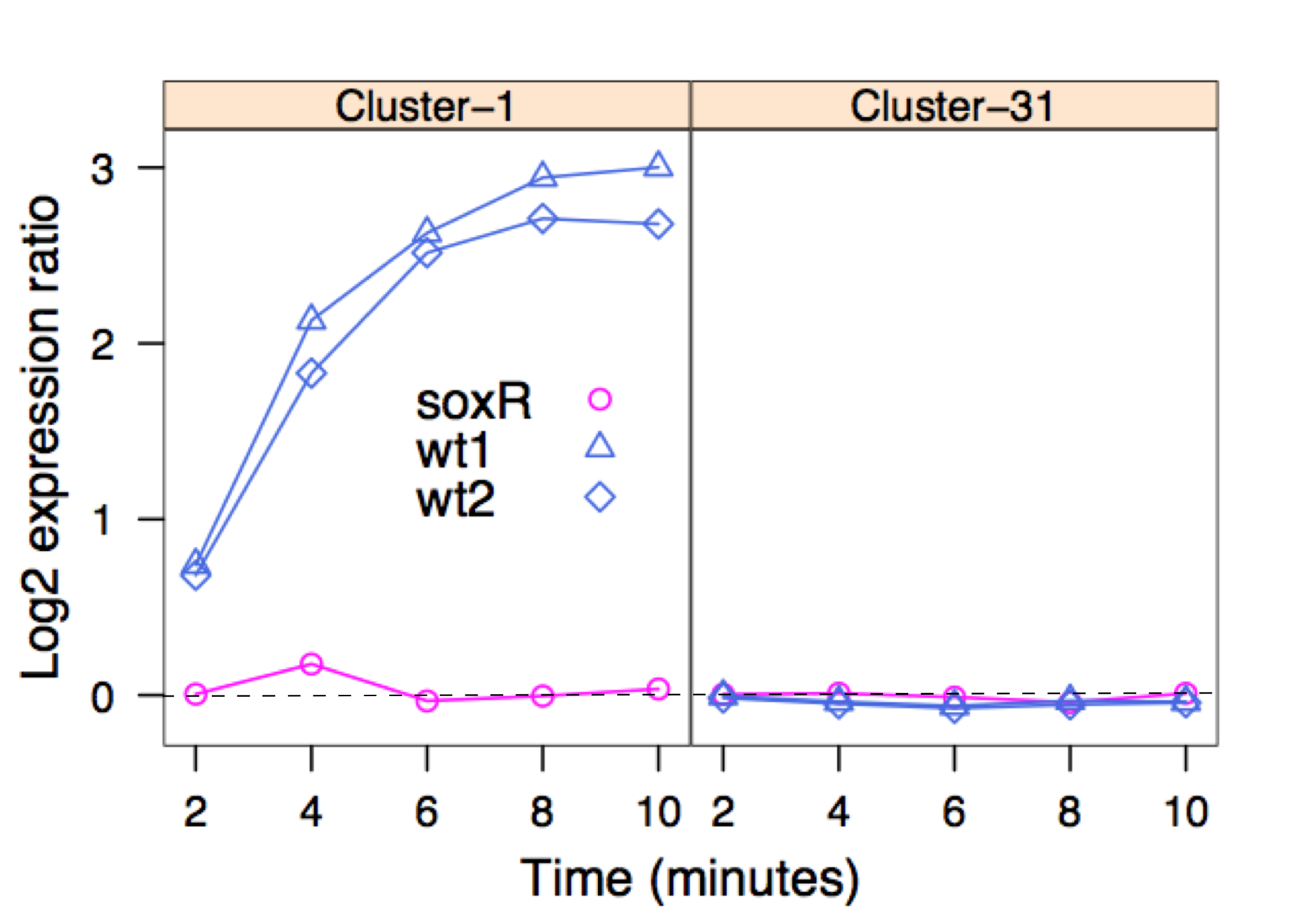

Supplement: Supplemental Figure S1 — Growth of strain MG1655 exposed to PQ. A culture of E. coli strain MG1665 (wt) was started by dilution of an overnight culture 1/100 in fresh EZ medium. The culture was grown at 37°C with strong aeration (250 rpm). At time = 0, the culture was split and one half was left untreated, while the other half was exposed to 500 µM paraquat. (0.70 MB TIF) [file pone.0001186.s001.tif]
