# Supplementary material for: Rapid Changes in Gene Expression Dynamics in Response to Superoxide Reveal SoxRS-Dependent and Independent Transcriptional Networks
Source: PLoS One. 2007 Nov 14;2(11):e1186. doi: 10.1371/journal.pone.0001186 (PMC2064960; doi:10.1371/journal.pone.0001186)
Supplement: Supplemental Table S1 — List of genes from the clusters in Figure 3. (0.68 MB DOC) [file pone.0001186.s003.doc]

Supplemental Table S1 – List of genes from the clusters in Figure 3.

| Cluster | Transcript ID | Gene | Description |
| --- | --- | --- | --- |
|  | (Array Design) | Name |  |
| 1 | IG_651167_651457-f | NO_MATCH | Intergenic |
| 1 | MG1655_yceE_b1053 | mdtG | YceE drug MFS transporter |
| 1 | MG1655_b1377 | ompN | outer membrane pore protein N, non-specific |
| 1 | IG_1434918_1435144-r | NO_MATCH | Intergenic |
| 1 | MG1655_tke8_b4427 | micC | MicC small RNA |
| 1 | MG1655_ydbK_b1378 | ydbK | putative pyruvate synthase |
| 1 | MG1655_b1451 | yncD | probable TonB-dependent receptor |
| 1 | MG1655_b1463 | nhoA | N-hydroxyarylamine O-acetyltransferase |
| 1 | IG_1619063_1619355-r | NO_MATCH | Intergenic |
| 1 | MG1655_fumC_b1611 | fumC | fumarase C monomer |
| 1 | EDL933_Z2873 | yobH | predicted protein |
| 1 | MG1655_nfo_b2159 | nfo | endonuclease IV |
| 1 | MG1655_yeiI_b2160 | yeiI | putative kinase |
| 1 | MG1655_micF_b4439 | micF | MicF RNA; antisense negative regulator of OmpF abundance |
| 1 | MG1655_inaA_b2237 | inaA | pH-inducible protein involved in stress response |
| 1 | MG1655_frvX_b3898 | frvX | frv operon protein |
| 1 | MG1655_kdgT_b3909 | kdgT | KdgT 2-keto-3-deoxygluconate transporter |
| 1 | MG1655_fpr_b3924 | fpr | flavodoxin NADP+ reductase |
| 1 | MG1655_soxS_b4062 | soxS | SoxS transcriptional activator |
| 1 | MG1655_yjjI_b4380 | yjjI | conserved hypothetical protein |
|  |  |  |  |
| 2 | MG1655_yadM_b0138 | yadM | predicted fimbrial-like adhesin protein |
| 2 | MG1655_yadR_b0156 | yadR | conserved hypothetical protein |
| 2 | MG1655_map_b0168 | map | methionine aminopeptidase |
| 2 | EDL933_ybaO_Z0555 | ybaO | putative LRP-like transcriptional regulator |
| 2 | MG1655_mdlA_b0448 | mdlA | predicted multidrug transporter subunit of ABC superfamily: ATP-binding component |
| 2 | MG1655_mdlB_b0449 | mdlB | predicted multidrug transporter subunit of ABC superfamily: ATP-binding component |
| 2 | MG1655_ybaL_b0478 | ybaL | YbaL CPA2 transporter |
| 2 | MG1655_ybdO_b0603 | ybdO | predicted DNA-binding transcriptional regulator LYSR-type |
| 2 | IG_709870_710157-f | NO_MATCH | Intergenic |
| 2 | MG1655_fldA_b0684 | fldA | oxidized flavodoxin 1 |
| 2 | MG1655_b0762 | ybhT | hypothetical protein |
| 2 | MG1655_ybjC_b0850 | ybjC | predicted inner membrane protein |
| 2 | MG1655_b0851 | nfsA | NADPH nitroreductase |
| 2 | MG1655_rimK_b0852 | rimK | ribosomal protein S6 modification protein |
| 2 | MG1655_poxB_b0871 | poxB | pyruvate oxidase monomer |
| 2 | MG1655_pqiA_b0950 | pqiA | paraquat-inducible protein A |
| 2 | MG1655_pqiB_b0951 | pqiB | paraquat-inducible protein B |
| 2 | MG1655_ymbA_b0952 | ymbA | conserved protein |
| 2 | MG1655_b1047 | mdoC | protein required for succinyl modification of osmoregulated periplasmic glucans |
| 2 | MG1655_acnA_b1276 | acnA | aconitase |
| 2 | MG1655_ribA_b1277 | ribA | GTP cyclohydrolase II |
| 2 | IG_1434918_1435144-f | NO_MATCH | Intergenic |
| 2 | SAKAI_ECs5181 | NO_MATCH | hypothetical protein |
| 2 | EDL933_Z2249 | nhoA | N-hydroxyarylamine O-acetyltransferase |
| 2 | MG1655_marA_b1531 | marA | MarA transcriptional activator |
| 2 | MG1655_b1533 | eamA | O-acetylserine/cysteine export protein |
| 2 | MG1655_b1534 | ydeE | YdeE MFS transporter |
| 2 | IG_1932629_1932862-r | NO_MATCH | Intergenic |
| 2 | MG1655_zwf_b1852 | zwf | glucose 6-phosphate-1-dehydrogenase |
| 2 | EDL933_Z3676 | ypeB | predicted protein |
| 2 | MG1655_b2411 | ligA | DNA ligase |
| 2 | MG1655_b2889 | idi | isopentenyl diphosphate isomerase |
| 2 | MG1655_fldB_b2895 | fldB | oxidized flavodoxin 2 |
| 2 | MG1655_ygfZ_b2898 | ygfZ | predicted folate-dependent regulatory protein |
| 2 | MG1655_yggX_b2962 | yggX | protein that protects iron-sulfur proteins against oxidative damage |
| 2 | MG1655_mltC_b2963 | mltC | membrane-bound lytic murein transglycosylase C |
| 2 | MG1655_ygiA_b3036 | ygiA | predicted protein |
| 2 | MG1655_yhbW_b3160 | yhbW | conserved protein |
| 2 | MG1655_yrbL_b3207 | yrbL | predicted protein |
| 2 | MG1655_yhcN_b3238 | yhcN | conserved hypothetical protein |
| 2 | MG1655_treF_b3519 | treF | trehalase, cytoplasmic |
| 2 | MG1655_yhjW_b3546 | eptB | phosphoethanolamine transferase |
| 2 | MG1655_yicM_b3662 | nepI | NepI MFS ribonucleoside transporter |
| 2 | MG1655_frvR_b3897 | frvR | FrvR predicted transcriptional regulator |
| 2 | MG1655_sodA_b3908 | sodA | superoxide dismutase (Mn) |
| 2 | MG1655_yjcB_b4060 | yjcB | conserved hypothetical protein |
| 2 | MG1655_yjjW_b4379 | yjjW | predicted pyruvate formate lyase activating enzyme |
|  |  |  |  |
| 3 | IG_70049_70386-r | NO_MATCH | Intergenic |
| 3 | MG1655_lpxC_b0096 | lpxC | UDP-3-O-acyl-N-acetylglucosamine deacetylase |
| 3 | MG1655_glnD_b0167 | glnD | uridylyltransferase / uridylyl-removing enzyme |
| 3 | MG1655_yagR_b0284 | yagR | putative molybdemum cofactor-binding oxidoreductase |
| 3 | MG1655_yagS_b0285 | yagS | putative oxidoreductase, FAD-binding domain |
| 3 | EDL933_lacA_Z0438 | lacA | galactoside O-acetyltransferase monomer |
| 3 | MG1655_hemB_b0369 | hemB | porphobilinogen synthase |
| 3 | CFT073_yaiH_c0480 | ampH | penicillin-binding protein |
| 3 | MG1655_yaiA_b0389 | yaiA | predicted protein |
| 3 | IG_406395_406651-f | NO_MATCH | Intergenic |
| 3 | MG1655_apbA_b0425 | panE | 2-dehydropantoate reductase |
| 3 | IG_450835_451293-f | NO_MATCH | Intergenic |
| 3 | IG_479933_480477-r | NO_MATCH | Intergenic |
| 3 | EDL933_acrB_Z0576 | acrB | AcrB RND-type permease |
| 3 | MG1655_acrA_b0463 | acrA | AcrA Membrane Fusion Protein |
| 3 | MG1655_b0572 | cusC | outer membrane factor of the CusCFBA copper efflux system |
| 3 | MG1655_nfnB_b0578 | nfnB | dihydropteridine reductase |
| 3 | MG1655_fur_b0683 | fur | Intergenic |
| 3 | IG_709870_710157-r | NO_MATCH |  |
| 3 | MG1655_cmr_b0842 | cmr | MdfA/Cmr MFS multidrug transporter |
| 3 | MG1655_ybjH_b0843 | ybjH | predicted protein |
| 3 | MG1655_ybjN_b0853 | ybjN | predicted oxidoreductase |
| 3 | IG_892657_893006-f | NO_MATCH | Intergenic |
| 3 | MG1655_artQ_b0862 | artQ | arginine ABC transporter |
| 3 | MG1655_artI_b0863 | artI | arginine ABC transporter |
| 3 | MG1655_artP_b0864 | artP | arginine ABC transporter |
| 3 | MG1655_cmk_b0910 | cmk | cytidylate kinase |
| 3 | MG1655_pncB_b0931 | pncB | nicotinate phosphoribosyltransferase |
| 3 | IG_1014683_1014937-f | NO_MATCH | Intergenic |
| 3 | MG1655_msyB_b1051 | msyB | acidic protein that suppresses mutants lacking function of protein export |
| 3 | IG_1115806_1116029-r | NO_MATCH | Intergenic |
| 3 | MG1655_ycgZ_b1164 | ycgZ | hypothetical protein |
| 3 | MG1655_ymgA_b1165 | ymgA | hypothetical protein |
| 3 | MG1655_ymgB_b1166 | ymgB | hypothetical protein |
| 3 | MG1655_ymgC_b1167 | ymgC | hypothetical protein |
| 3 | IG_1216220_1216508-f | NO_MATCH | Intergenic |
| 3 | MG1655_b1168 | ycgG | conserved protein |
| 3 | MG1655_ychA_b1214 | ychA | predicted transcriptional regulator |
| 3 | MG1655_chaC_b1218 | chaC | cation transport regulator |
| 3 | IG_1308294_1308592-f | NO_MATCH | Intergenic |
| 3 | MG1655_ycjX_b1321 | ycjX | conserved protein |
| 3 | MG1655_feaB_b1385 | feaB | phenylacetaldehyde dehydrogenase |
| 3 | MG1655_b1431 | ydcL | conserved hypothetical protein |
| 3 | MG1655_b1438 | ydcQ | hypothetical protein |
| 3 | CFT073_c1862 | ydcQ | hypothetical protein |
| 3 | MG1655_marR_b1530 | marR | MarR transcriptional repressor |
| 3 | MG1655_marB_b1532 | marB | multiple antibiotic resistance protein |
| 3 | IG_1619063_1619355-f | NO_MATCH | Intergenic |
| 3 | MG1655_rydB_b4430 | rydB | small RNA regulator of RpoS |
| 3 | MG1655_b1746 | astD | aldehyde dehydrogenase |
| 3 | IG_1906791_1907331-f | NO_MATCH | Intergenic |
| 3 | IG_1934339_1934675-r | NO_MATCH | Intergenic |
| 3 | IG_2311197_2311507-f | NO_MATCH | Intergenic |
| 3 | MG1655_b2299 | yfcD | putative enzyme (Nudix hydrolase) |
| 3 | CFT073_c3010 | NO_MATCH | hypothetical protein |
| 3 | MG1655_yfhD_b2558 | yfhD | predicted transglycosylase |
| 3 | MG1655_b2680 | ygaX | predicted transporter |
| 3 | MG1655_b2681 | ygaY | predicted transporter |
| 3 | MG1655_iap_b2753 | iap | alkaline phosphatase isozyme conversion protein |
| 3 | MG1655_tolC_b3035 | tolC | TolC outer membrane channel |
| 3 | MG1655_yraL_b3146 | yraL | predicted methyltransferase |
| 3 | MG1655_yrbI_b3198 | kdsC | 3-deoxy-D-<i>manno</i>-octulosonate 8-phosphate phosphatase monomer |
| 3 | MG1655_yrbK_b3199 | yrbK | conserved protein |
| 3 | MG1655_yhbN_b3200 | lptA | YhbG/YhbN ABC transporter |
| 3 | MG1655_yhbG_b3201 | lptB | YhbG/YhbN ABC transporter |
| 3 | MG1655_envZ_b3404 | envZ | EnvZ |
| 3 | MG1655_ompR_b3405 | ompR | CPLX0-5729 |
| 3 | MG1655_rpoH_b3461 | rpoH | sigma32 factor |
| 3 | IG_3635041_3635271-r | NO_MATCH | Intergenic |
| 3 | MG1655_yhjX_b3547 | yhjX | YhjX MFS transporter |
| 3 | MG1655_rfaY_b3625 | rfaY | lipopolysaccharide core biosynthesis protein |
| 3 | MG1655_dgoT_b3691 | dgoT | YidT galactonate MFS transporter |
| 3 | MG1655_pgi_b4025 | pgi | phosphoglucose isomerase |
| 3 | MG1655_b4140 | fxsA | inner membrane protein; overproduction inhibits F exclusion of bacteriophage T7 |
|  |  |  |  |
| 4 | IG_11787_12162-f | NO_MATCH | Intergenic |
| 4 | MG1655_dnaK_b0014 | dnaK | chaperone Hsp70; DNA biosynthesis; autoregulated heat shock proteins |
| 4 | MG1655_dnaJ_b0015 | dnaJ | chaperone with DnaK; heat shock protein |
| 4 | MG1655_yacA_b0097 | secM | secretion monitor that regulates SecA translation (General Secretory Pathway) |
| 4 | MG1655_secA_b0098 | secA | Sec Protein Secretion Complex |
| 4 | MG1655_pdhR_b0113 | pdhR | PdhR transcriptional dual regulator |
| 4 | MG1655_yadK_b0136 | yadK | predicted fimbrial-like adhesin protein |
| 4 | MG1655_yadL_b0137 | yadL | predicted fimbrial-like adhesin protein |
| 4 | MG1655_dgt_b0160 | dgt | deoxyguanosinetriphosphate triphosphohydrolase |
| 4 | MG1655_b0221 | fadE | acyl-CoA dehydrogenase |
| 4 | IG_292173_292443-r | NO_MATCH | Intergenic |
| 4 | IG_296321_296604-r | NO_MATCH | Intergenic |
| 4 | MG1655_yagP_b0282 | yagP | predicted transcriptional regulator LYSR-type |
| 4 | MG1655_yagQ_b0283 | yagQ | conserved protein |
| 4 | MG1655_yagW_b0290 | yagW | predicted receptor |
| 4 | MG1655_tauA_b0365 | tauA | TauA/TauB/TauC ABC transporter |
| 4 | MG1655_tauC_b0367 | tauC | TauA/TauB/TauC ABC transporter |
| 4 | MG1655_b0370 | ykiB | predicted protein |
| 4 | CFT073_c0507 | NO_MATCH | hypothetical protein |
| 4 | MG1655_dxs_b0420 | dxs | 1-deoxyxylulose-5-phosphate synthase |
| 4 | MG1655_xseB_b0422 | xseB | exonuclease VII, small subunit |
| 4 | MG1655_apt_b0469 | apt | adenine phosphoribosyltransferase |
| 4 | MG1655_htpG_b0473 | htpG | HtpG monomer |
| 4 | MG1655_ylcC_b0573 | cusF | periplasmic copper-binding protein |
| 4 | MG1655_citA_b0619 | citA | sensory histidine kinase in two-component regulatory system with CitB |
| 4 | MG1655_citB_b0620 | citB | DNA-binding response regulator in two-component regulatory system with CitA |
| 4 | MG1655_lipA_b0628 | lipA | lipoate synthase monomer |
| 4 | MG1655_ybeD_b0631 | ybeD | conserved protein |
| 4 | MG1655_ybeY_b0659 | ybeY | conserved protein |
| 4 | MG1655_ybfF_b0686 | ybfF | esterase |
| 4 | MG1655_modE_b0761 | modE | CPLX0-5664 |
| 4 | MG1655_ybhA_b0766 | ybhA | pyridoxal phosphatase / fructose 1,6-bisphosphatase |
| 4 | CFT073_bioB_c0855 | bioB | biotin synthase monomer |
| 4 | IG_836660_836887-r | NO_MATCH | Intergenic |
| 4 | IG_892657_893006-r | NO_MATCH | Intergenic |
| 4 | IG_899799_900088-r | NO_MATCH | Intergenic |
| 4 | MG1655_artM_b0861 | artM | arginine ABC transporter |
| 4 | MG1655_ybjP_b0865 | ybjP | predicted lipoprotein |
| 4 | CFT073_c1016 | macB | MacAB macrolide efflux transporter complex |
| 4 | MG1655_htrB_b1054 | lpxL | lauroyl acyltransferase |
| 4 | MG1655_b1173 | ycgI | hypothetical protein |
| 4 | MG1655_b1213 | ychQ | predicted transcriptional regulator |
| 4 | MG1655_kdsA_b1215 | kdsA | 3-deoxy-D-<i>manno</i>-octulosonic acid 8-phosphate synthase |
| 4 | MG1655_aldH_b1300 | puuC | &gamma;-glutamyl-&gamma;-aminobutyraldehyde dehydrogenase |
| 4 | MG1655_ordL_b1301 | puuB | &gamma;-glutamylputrescine oxidase |
| 4 | MG1655_ycjF_b1322 | ycjF | putative membrane protein |
| 4 | MG1655_b1559 | ydfT | Qin prophage; predicted antitermination protein Q |
| 4 | MG1655_b1688 | ydiK | hypothetical protein; transcription may be purine regulated |
| 4 | MG1655_htpX_b1829 | htpX | heat shock protein, integral membrane protein |
| 4 | IG_2228406_2228643-r | NO_MATCH | Intergenic |
| 4 | MG1655_b2170 | setB | SetB MFS transporter |
| 4 | MG1655_yeiR_b2173 | yeiR | predicted enzyme |
| 4 | IG_2304775_2304991-f | NO_MATCH | Intergenic |
| 4 | MG1655_yfcE_b2300 | yfcE | phosphodiesterase |
| 4 | MG1655_b2335 | yfcR | putative fimbrial protein |
| 4 | MG1655_yfcS_b2336 | yfcS | putative chaperone |
| 4 | MG1655_clpB_b2592 | clpB | ClpB chaperone |
| 4 | MG1655_ypjF_b2646 | ypjF | CP4-57 prophage; toxin of the YpjF-YfjZ toxin-antitoxin system |
| 4 | MG1655_b1173 | ypjA | adhesin-like autotransporter |
| 4 | IG_2885243_2885600-r | NO_MATCH | Intergenic |
| 4 | MG1655_syd_b2793 | syd | predicted protein |
| 4 | MG1655_yggS_b2951 | yggS | predicted enzyme |
| 4 | MG1655_nupG_b2964 | nupG | NupG nucleoside MFS transporter |
| 4 | MG1655_mdaB_b3028 | mdaB | NADPH quinone reductase |
| 4 | MG1655_ygiB_b3037 | ygiB | conserved outer membrane protein |
| 4 | MG1655_ribB_b3041 | ribB | 3,4-dihydroxy-2-butanone 4-phosphate synthase |
| 4 | CFT073_c3818 | NO_MATCH | hypothetical protein |
| 4 | IG_3214133_3214419-f | NO_MATCH | Intergenic |
| 4 | IG_3237188_3237583-r | NO_MATCH | Intergenic |
| 4 | MG1655_yraK_b3145 | yraK | putative fimbrial protein |
| 4 | EDL933_Z4504 | yraK | putative fimbrial protein |
| 4 | MG1655_rpoN_b3202 | rpoN | sigma54 factor |
| 4 | MG1655_acrE_b3265 | acrE | transmembrane protein affects septum formation and cell membrane permeability |
| 4 | MG1655_yhdN_b3293 | yhdN | conserved protein |
| 4 | MG1655_b3400 | hslR | heat shock protein Hsp15 |
| 4 | MG1655_b3401 | hslO | molecular chaperone Hsp33 |
| 4 | IG_3543836_3544194-f | NO_MATCH | Intergenic |
| 4 | MG1655_yhhA_b3448 | yhhA | conserved protein |
| 4 | MG1655_yhiQ_b3497 | yhiQ | predicted SAM-dependent methyltransferase |
| 4 | MG1655_prlC_b3498 | prlC | oligopeptidase A |
| 4 | IG_3666819_3667221-f | NO_MATCH | Intergenic |
| 4 | MG1655_yhjB_b3520 | yhjB | putative regulator |
| 4 | IG_3669525_3669971-f | NO_MATCH | Intergenic |
| 4 | MG1655_yhjJ_b3527 | yhjJ | putative peptidase |
| 4 | MG1655_aldB_b3588 | aldB | acetaldehyde dehydrogenase |
| 4 | MG1655_mutM_b3635 | mutM | formamidopyrimidine DNA glycosylase |
| 4 | MG1655_asnA_b3744 | asnA | aspartate-ammonia ligase |
| 4 | MG1655_yieP_b3755 | yieP | predicted transcriptional regulator |
| 4 | CFT073_yiiL_c4850 | yiiL | L-rhamnose mutarotase |
| 4 | MG1655_hslU_b3931 | hslU | ATPase component of the HslVU protease |
| 4 | MG1655_hslV_b3932 | hslV | peptidase component of the HslVU protease |
| 4 | MG1655_yjiC_b4325 | yjiC | hypothetical protein |
| 4 | MG1655_deoB_b4383 | deoB | phosphopentomutase |
|  |  |  |  |
| 5 | MG1655_fepA_b0584 | fepA | FepA, outer membrane receptor for ferric enterobactin (enterochelin) and colicins B and D |
| 5 | MG1655_fes_b0585 | fes | enterochelin esterase |
| 5 | EDL933_Z0726 | ybdZ | conserved protein |
| 5 | MG1655_entF_b0586 | entF | serine activating enzyme |
| 5 | MG1655_ybdA_b0591 | entS | EntS MFS transporter |
| 5 | MG1655_entC_b0593 | entC | isochorismate synthase, enterobactin specific |
| 5 | MG1655_entE_b0594 | entE | enterobactin synthase multienzyme complex |
| 5 | MG1655_entB_b0595 | entB | apo-EntB |
| 5 | MG1655_ybiX_b0804 | ybiX | conserved protein |
| 5 | MG1655_b0805 | fiu | putative outer membrane receptor for iron transport |
| 5 | IG_840755_841018-r | NO_MATCH | Intergenic |
| 5 | MG1655_fhuE_b1102 | fhuE | outer membrane receptor for ferric iron uptake |
| 5 | MG1655_b1684 | sufA | Fe-S cluster assembly protein |
| 5 | MG1655_cirA_b2155 | cirA | outer membrane receptor for iron-regulated colicin I receptor; porin; requires tonB gene product |
| 5 | IG_2244790_2245082-r | NO_MATCH | Intergenic |
| 5 | MG1655_nrdH_b2673 | nrdH | glutaredoxin-like protein |
| 5 | MG1655_nrdI_b2674 | nrdI | conserved protein that may stimulate ribonucleotide reductase |
| 6 | MG1655_fhuA_b0150 | fhuA | FhuA outer membrane protein receptor for ferrichrome, colicin M, and phages T1, T5, and phi80 |
| 6 | MG1655_fhuC_b0151 | fhuC | ferrichrome uptake system |
| 6 | MG1655_fhuD_b0152 | fhuD | ferrichrome uptake system |
| 6 | MG1655_fhuB_b0153 | fhuB | ferrichrome uptake system |
| 6 | MG1655_entD_b0583 | entD | phosphopantetheinyl transferase |
| 6 | IG_611718_612037-r | NO_MATCH | Intergenic |
| 6 | CFT073_c0670 | NO_MATCH | hypothetical protein |
| 6 | IG_613163_613379-r | NO_MATCH | Intergenic |
| 6 | MG1655_fepC_b0588 | fepC | Ferric Enterobactin Transport System |
| 6 | MG1655_fepG_b0589 | fepG | Ferric Enterobactin Transport System |
| 6 | MG1655_fepD_b0590 | fepD | Ferric Enterobactin Transport System |
| 6 | MG1655_fepB_b0592 | fepB | Ferric Enterobactin Transport System |
| 6 | IG_623734_624107-r | NO_MATCH | Intergenic |
| 6 | MG1655_entA_b0596 | entA | 2,3-dihydro-2,3-dihydroxybenzoate dehydrogenase |
| 6 | MG1655_ybdB_b0597 | ybdB | esterase |
| 6 | IG_837149_837412-r | NO_MATCH | Intergenic |
| 6 | MG1655_ybiI_b0803 | ybiI | conserved protein |
| 6 | MG1655_b1017 | ycdN | conserved protein |
| 6 | MG1655_ycdO_b1018 | ycdO | conserved protein |
| 6 | MG1655_ycdB_b1019 | ycdB | conserved protein |
| 6 | MG1655_tonB_b1252 | tonB | TonB protein; energy transducer; uptake of iron, cyanocobalimin; sensitivity to phages, colicins |
| 6 | MG1655_b1452 | yncE | conserved protein |
| 6 | MG1655_b1679 | sufE | sulfur acceptor that activates SufS cysteine desulfurase |
| 6 | MG1655_sufS_b1680 | sufS | L-selenocysteine lyase (and L-cysteine desulfurase) monomer |
| 6 | MG1655_b1681 | sufD | component of SufB-SufC-SufD cysteine desulfurase (SufS) activator complex |
| 6 | MG1655_b1682 | sufC | component of SufBCD complex |
| 6 | MG1655_b1683 | sufB | component of SufB-SufC-SufD cysteine desulfurase (SufS) activator complex |
| 6 | MG1655_ydiE_b1705 | ydiE | conserved protein |
| 6 | MG1655_nrdE_b2675 | nrdE | ribonucleoside-diphosphate reductase 2 |
| 6 | MG1655_nrdF_b2676 | nrdF | ribonucleoside-diphosphate reductase 2 |
| 6 | MG1655_b3337 | bfd | bacterioferritin-associated ferredoxin |
| 6 | MG1655_ibpB_b3686 | ibpB | small heat shock protein IbpB |
| 6 | MG1655_fecR_b4292 | fecR | regulator for fec operon, periplasmic |
| 6 | MG1655_fecI_b4293 | fecI | RNA polymerase, sigma19 factor |
| 6 | MG1655_fhuF_b4367 | fhuF | acts in reduction of ferrioxamine B iron |
|  |  |  |  |
| 7 | MG1655_gcd_b0124 | gcd | glucose dehydrogenase |
| 7 | IG_167265_167483-r | NO_MATCH | Intergenic |
| 7 | MG1655_thiJ_b0424 | yajL | conserved protein |
| 7 | MG1655_ybaN_b0468 | ybaN | hypothetical protein |
| 7 | IG_638732_638945-f | NO_MATCH | Intergenic |
| 7 | CFT073_ahpF_c0695 | ahpF | AhpF component |
| 7 | MG1655_ybhB_b0773 | ybhB | predicted kinase inhibitor |
| 7 | MG1655_bioA_b0774 | bioA | adenosylmethionine-8-amino-7-oxononanoate aminotransferase monomer |
| 7 | MG1655_bioF_b0776 | bioF | 8-amino-7-oxononanoate synthase |
| 7 | MG1655_bioD_b0778 | bioD | dethiobiotin synthetase subunit |
| 7 | IG_812171_812748-f | NO_MATCH | Intergenic |
| 7 | IG_812171_812748-r | NO_MATCH | Intergenic |
| 7 | MG1655_ybiJ_b0802 | ybiJ | predicted protein |
| 7 | IG_840755_841018-f | NO_MATCH | Intergenic |
| 7 | MG1655_dps_b0812 | dps | stationary phase nucleoid protein that sequesters iron and protects DNA from damage |
| 7 | IG_1080037_1080569-r | NO_MATCH | Intergenic |
| 7 | IG_1083871_1084214-f | NO_MATCH | Intergenic |
| 7 | MG1655_yceP_b1060 | bssS | regulator of biofilm formation |
| 7 | SAKAI_ECs1874 | puuA | &gamma;-glutamylputrescine synthetase |
| 7 | MG1655_ydcE_b1461 | pptA | probable 4-oxalocrotonate tautomerase (4-OT) monomer |
| 7 | MG1655_yddB_b1495 | yddB | conserved protein |
| 7 | MG1655_yddA_b1496 | yddA | YddA complex |
| 7 | MG1655_b1995 | NO_MATCH | hypothetical protein |
| 7 | MG1655_yegQ_b2081 | yegQ | hypothetical protein |
| 7 | MG1655_ryeE_b4438 | ryeE | small RNA that interacts with Hfq |
| 7 | MG1655_yohM_b2106 | rcnA | membrane protein conferring nickel and cobalt resistance |
| 7 | MG1655_yojI_b2211 | yojI | YojI |
| 7 | IG_2539274_2539698-r | NO_MATCH | Intergenic |
| 7 | MG1655_sseB_b2522 | sseB | overproduction causes enhanced serine sensitivity |
| 7 | CFT073_pepB_c3048 | pepB | aminopeptidase B |
| 7 | MG1655_yfhJ_b2524 | iscX | protein with possible role in iron-sulfur cluster biogenesis |
| 7 | MG1655_fdx_b2525 | fdx | oxidized ferredoxin |
| 7 | MG1655_hscA_b2526 | hscA | chaperone, member of Hsp70 protein family |
| 7 | MG1655_b2527 | hscB | Hsc20 co-chaperone that acts with Hsc66 in IscU iron-sulfur cluster assembly |
| 7 | MG1655_b2528 | iscA | iron-sulfur cluster assembly protein |
| 7 | MG1655_b2529 | iscU | scaffold protein involved in iron-sulfur cluster assembly |
| 7 | MG1655_iscS_b2530 | iscS | cysteine desulfurase monomer |
| 7 | MG1655_iscR_b2531 | iscR | IscR transcriptional regulator |
| 7 | MG1655_trxC_b2582 | trxC | oxidized thioredoxin 2 |
| 7 | IG_2798497_2798743-r | NO_MATCH | Intergenic |
| 7 | IG_2802483_2802835-f | NO_MATCH | Intergenic |
| 7 | IG_2889922_2890236-r | NO_MATCH | Intergenic |
| 7 | MG1655_exbD_b3005 | exbD | ExbD uptake of enterochelin; tonB-dependent uptake of B colicins |
| 7 | MG1655_exbB_b3006 | exbB | ExbB protein; uptake of enterochelin; tonB-dependent uptake of B colicins |
| 7 | MG1655_yqjH_b3070 | yqjH | hypothetical protein |
| 7 | MG1655_yhaM_b3108 | yhaM | conserved protein |
| 7 | MG1655_yhaN_b3109 | yhaN | conserved protein |
| 7 | MG1655_yhaO_b3110 | yhaO | YhaO STP transporter |
| 7 | IG_3255598_3255927-r | NO_MATCH | Intergenic |
| 7 | MG1655_bfr_b3336 | bfr | bacterioferritin monomer |
| 7 | MG1655_feoA_b3408 | feoA | ferrous iron transport protein A |
| 7 | MG1655_ryhB_b4451 | ryhB | regulatory antisense RNA involved in iron homeostasis |
| 7 | IG_3708138_3708427-r | NO_MATCH | Intergenic |
| 7 | MG1655_ibpA_b3687 | ibpA | small heat shock protein IbpA |
| 7 | MG1655_katG_b3942 | katG | hydroperoxidase I |
| 7 | MG1655_fecA_b4291 | fecA | outer membrane receptor; citrate-dependent iron transport, outer membrane receptor |
| 7 | MG1655_bglJ_b4366 | bglJ | BglJ transcriptional regulator |
|  |  |  |  |
| 8 | MG1655_grxA_b0849 | grxA | oxidized glutaredoxin 1 |
| 8 | MG1655_b1729 | ydjN | predicted transporter |
| 8 | MG1655_yeeD_b2012 | yeeD | conserved hypothetical protein |
| 8 | MG1655_yeeE_b2013 | yeeE | putative transport system permease protein |
| 8 | IG_2165052_2165133-r | NO_MATCH | Intergenic |
| 8 | MG1655_cysA_b2422 | cysA | sulfate ABC transporter |
| 8 | MG1655_cysW_b2423 | cysW | sulfate ABC transporter |
| 8 | MG1655_cysU_b2424 | cysU | sulfate ABC transporter |
| 8 | MG1655_cysP_b2425 | cysP | thiosulfate ABC transporter |
| 8 | MG1655_cysC_b2750 | cysC | adenylylsulfate kinase |
| 8 | MG1655_cysN_b2751 | cysN | sulfate adenylyltransferase |
| 8 | MG1655_cysD_b2752 | cysD | sulfate adenylyltransferase |
| 8 | MG1655_cysH_b2762 | cysH | 3'-phospho-adenylylsulfate reductase |
| 8 | MG1655_cysI_b2763 | cysI | sulfite reductase hemoprotein subunit |
| 8 | MG1655_cysJ_b2764 | cysJ | sulfite reductase flavoprotein subunit |
| 8 | MG1655_sbp_b3917 | sbp | sulfate ABC transporter |
| 8 | MG1655_oxyS_b4458 | oxyS | OxyS RNA; oxidative stress regulator |
|  |  |  |  |
| 9 | MG1655_thrL_b0001 | thrL | thr operon leader peptide |
| 9 | MG1655_thrB_b0003 | thrB | homoserine kinase |
| 9 | MG1655_thrC_b0004 | thrC | threonine synthase |
| 9 | MG1655_leuD_b0071 | leuD | isopropylmalate isomerase |
| 9 | MG1655_leuA_b0074 | leuA | 2-isopropylmalate synthase |
| 9 | MG1655_leuL_b0075 | leuL | leu operon leader peptide |
| 9 | MG1655_yafK_b0224 | yafK | conserved protein |
| 9 | IG_578117_578406-r | NO_MATCH | Intergenic |
| 9 | MG1655_ybcW_b0559 | ybcW | hypothetical protein |
| 9 | IG_579310_580056-f | NO_MATCH | Intergenic |
| 9 | MG1655_appY_b0564 | appY | AppY transcriptional activator |
| 9 | MG1655_ybjE_b0874 | ybjE | putative surface protein |
| 9 | MG1655_ompF_b0929 | ompF | outer membrane porin OmpF |
| 9 | MG1655_ndh_b1109 | ndh | NADH dehydrogenase II / NADH cupric reductase |
| 9 | IG_1166613_1166821-f | NO_MATCH | Intergenic |
| 9 | MG1655_mcrA_b1159 | mcrA | restriction of DNA at 5-methylcytosine residues |
| 9 | MG1655_ycgW_b1160 | elbA | predicted protein |
| 9 | MG1655_b1541 | ydfZ | conserved protein |
| 9 | MG1655_yeaS_b1798 | leuE | leucine export protein |
| 9 | CFT073_c2307 | flhC | CPLX0-5722 |
| 9 | MG1655_yecG_b1895 | uspC | universal stress protein |
| 9 | MG1655_yodB_b1974 | yodB | predicted cytochrome |
| 9 | MG1655_yegH_b2063 | yegH | putative transport protein |
| 9 | IG_2137508_2137780-f | NO_MATCH | Intergenic |
| 9 | MG1655_pbpG_b2134 | pbpG | penicillin-binding protein 7 |
| 9 | MG1655_b2255 | arnA | UDP-L-Ara4N formyltransferase / UDP-GlcA C-4"-decarboxylase |
| 9 | MG1655_b2256 | yfbH | conserved protein |
| 9 | MG1655_b2290 | yfbQ | predicted aminotransferase |
| 9 | MG1655_b2504 | yfgG | predicted protein |
| 9 | IG_2627502_2627811-f | NO_MATCH | Intergenic |
| 9 | MG1655_xseA_b2509 | xseA | exonuclease VII, large subunit |
| 9 | MG1655_nadB_b2574 | nadB | L-aspartate oxidase |
| 9 | MG1655_ypjE_b2612 | yfjD | predicted inner membrane protein |
| 9 | MG1655_serA_b2913 | serA | 2-oxoglutarate reductase / phosphoglycerate dehydrogenase |
| 9 | MG1655_speC_b2965 | speC | ornithine decarboxylase, biosynthetic |
| 9 | IG_3351686_3352266-f | NO_MATCH | Intergenic |
| 9 | MG1655_hdeA_b3510 | hdeA | acid-resistance protein, possible chaperone |
| 9 | MG1655_gadX_b3516 | gadX | GadX transcriptional activator |
| 9 | CFT073_yidQ_c4608 | NO_MATCH | hypothetical protein |
| 9 | MG1655_pstA_b3726 | pstA | phosphate ABC transporter |
| 9 | CFT073_pstC_c4652 | pstC | phosphate ABC transporter |
| 9 | MG1655_glmS_b3729 | glmS | L-glutamine:D-fructose-6-phosphate aminotransferase |
| 9 | MG1655_rbsD_b3748 | rbsD | D-ribose utilization |
| 9 | MG1655_tyrB_b4054 | tyrB | aromatic-amino-acid transaminase |
| 9 | MG1655_basS_b4112 | basS | BasS-P<SUP>his</SUP> |
| 9 | MG1655_basR_b4113 | basR | BasR-Phosphorylated transcriptional regulator |
|  |  |  |  |
| 10 | MG1655_leuB_b0073 | leuB | 3-isopropylmalate dehydrogenase |
| 10 | CFT073_c2347 | nmpC | outer membrane porin protein; locus of qsr prophage |
| 10 | IG_769835_770677-r | NO_MATCH | Intergenic |
| 10 | MG1655_cydA_b0733 | cydA | cytochrome <i>bd</i>-I terminal oxidase subunit I |
| 10 | MG1655_cydB_b0734 | cydB | cytochrome <i>bd</i>-I terminal oxidase subunit II |
| 10 | SAKAI_ECs5391 | ybgT | conserved protein |
| 10 | MG1655_ybgE_b0735 | ybgE | conserved hypothetical protein |
| 10 | MG1655_ybiP_b0815 | ybiP | predicted hydrolase, inner membrane |
| 10 | MG1655_pflA_b0902 | pflA | pyruvate formate-lyase activating enzyme |
| 10 | MG1655_focA_b0904 | focA | FocA formate FNT transporter |
| 10 | CFT073_c1152 | NO_MATCH | hypothetical protein |
| 10 | MG1655_flgB_b1073 | flgB | flagellar basal-body rod protein FlgB |
| 10 | MG1655_flgC_b1074 | flgC | flagellar basal-body rod protein FlgC |
| 10 | IG_1197461_1197917-f | NO_MATCH | Intergenic |
| 10 | MG1655_hemA_b1210 | hemA | glutamyl-tRNA reductase |
| 10 | MG1655_prfA_b1211 | prfA | peptide chain release factor RF1 |
| 10 | MG1655_hemK_b1212 | prmC | protein-(glutamine-N5) methyltransferase |
| 10 | MG1655_tpr_b1229 | rttR | small RNA processed from <i>tyrT</i> transcript |
| 10 | MG1655_tpr_b1229 | tpr | protamine-like protein |
| 10 | MG1655_adhE_b1241 | adhE | PFL-deactivase / alcohol dehydrogenase / acetaldehyde dehydrogenase |
| 10 | MG1655_ydcH_b1426 | ydcH | conserved hypothetical protein |
| 10 | SAKAI_ECs5443 | fdnG | formate dehydrogenase N, &alpha; subunit |
| 10 | MG1655_b1600 | mdtJ | MdtJ SMR protein |
| 10 | MG1655_b1605 | ydgI | ArcD APC transporter |
| 10 | MG1655_ydiF_b1694 | ydiF | fused predicted acetyl-CoA:acetoacetyl-CoA transferase: &alpha; subunit/&beta; subunit |
| 10 | MG1655_b1756 | ynjD | YnjC/YnjD ABC transporter |
| 10 | IG_1903284_1903657-f | NO_MATCH | Intergenic |
| 10 | MG1655_b1821 | yebN | conserved inner membrane protein |
| 10 | MG1655_yecT_b1877 | yecT | hypothetical protein |
| 10 | MG1655_flhE_b1878 | flhE | conserved protein |
| 10 | MG1655_flhB_b1880 | flhB | flagellar biosynthesis protein FlhB |
| 10 | MG1655_flhC_b1891 | flhC | CPLX0-5722 |
| 10 | MG1655_flhD_b1892 | flhD | CPLX0-5722 |
| 10 | MG1655_ftn_b1905 | ftnA | cytoplasmic ferritin, an iron storage protein) |
| 10 | MG1655_fliA_b1922 | fliA | sigma28 factor |
| 10 | MG1655_fliF_b1938 | fliF | flagellar M-ring protein FliF; basal-body MS(membrane and supramembrane)-ring and collar protein |
| 10 | MG1655_fliG_b1939 | fliG | flagellar motor switch protein FliG |
| 10 | MG1655_fliL_b1944 | fliL | flagellar biosynthesis |
| 10 | MG1655_gatD_b2091 | gatD | galactitol-1-phosphate dehydrogenase |
| 10 | MG1655_gatC_b2092 | gatC | galactitol PTS permease |
| 10 | MG1655_gatA_b2094 | gatA | galactitol PTS permease |
| 10 | MG1655_gatZ_b2095 | gatZ | tagatose-1,6-bisphosphate aldolase 2 |
| 10 | MG1655_gatY_b2096 | gatY | tagatose-1,6-bisphosphate aldolase 2 |
| 10 | MG1655_fruA_b2167 | fruA | EIIFru |
| 10 | MG1655_fruK_b2168 | fruK | 1-phosphofructokinase monomer |
| 10 | MG1655_fruB_b2169 | fruB | EIIFru |
| 10 | MG1655_spr_b2175 | spr | predicted peptidase, outer membrane lipoprotein |
| 10 | MG1655_bcr_b2182 | bcr | Bcr multidrug MFS transporter |
| 10 | MG1655_napH_b2204 | napH | ferredoxin-type protein |
| 10 | MG1655_napA_b2206 | napA | large subunit of periplasmic nitrate reductase, molybdoprotein |
| 10 | MG1655_napF_b2208 | napF | ferredoxin-type protein |
| 10 | MG1655_hypB_b2727 | hypB | accessory protein for nickel incorporation into hydrogenase isoenzymes |
| 10 | MG1655_hypE_b2730 | hypE | hydrogenase maturation protein |
| 10 | MG1655_hybA_b2996 | hybA | hydrogenase 2 4Fe-4S ferredoxin-type component |
| 10 | MG1655_b2997 | hybO | hydrogenase 2, small subunit |
| 10 | CFT073_ygjT_c3846 | alx | putative membrane-bound redox modulator that is induced by high pH |
| 10 | MG1655_smg_b3284 | smg | conserved protein |
| 10 | MG1655_malT_b3418 | malT | MalT-MalK |
| 10 | CFT073_malT_c4196 | malT | MalT-MalK |
| 10 | MG1655_gntU_1_b3436 | gntU | GntU gluconate Gnt transporter |
| 10 | MG1655_yibO_b3612 | gpmI | phosphoglycerate mutase, cofactor independent |
| 10 | EDL933_ilvB_Z5165 | ilvB | IlvB / predicted 2-hydroxyglutarate synthase |
| 10 | MG1655_ivbL_b3672 | ivbL | ilvB operon leader peptide |
| 10 | MG1655_pstS_b3728 | pstS | phosphate ABC transporter |
| 10 | MG1655_rbsA_b3749 | rbsA | ribose ABC transporter |
| 10 | MG1655_rbsC_b3750 | rbsC | ribose ABC transporter |
| 10 | MG1655_ilvG_2_b3768 | b4488 | ilvG |
| 10 | MG1655_ilvC_b3774 | ilvC | acetohydroxy acid isomeroreductase |
| 10 | MG1655_ppc_b3956 | ppc | phosphoenolpyruvate carboxylase |
| 10 | MG1655_malE_b4034 | malE | Maltose/Maltodextrin Transport System |
| 10 | MG1655_dcuA_b4138 | dcuA | DcuA dicarboxylate Dcu transporter |
| 10 | MG1655_nrdD_b4238 | nrdD | ribonucleoside-triphosphate reductase |
| 10 | MG1655_b4256 | yjgM | predicted acetyltransferase |
|  |  |  |  |
| 11 | CFT073_c0406 | ykgO | predicted ribosomal protein |
| 11 | MG1655_ykgM_b0296 | ykgM | predicted ribosomal protein |
| 11 | IG_637857_638167-f | NO_MATCH | Intergenic |
| 11 | MG1655_gltA_b0720 | gltA | citrate synthase monomer |
| 11 | CFT073_c0797 | NO_MATCH | Intergenic |
| 11 | IG_761963_762236-r | NO_MATCH | Intergenic |
| 11 | MG1655_ybiK_b0828 | iaaA | &beta; cleavage product of IaaA |
| 11 | MG1655_b0829 | gsiA | gsiABCD glutathione ABC transporter |
| 11 | MG1655_b0830 | gsiB | gsiABCD glutathione ABC transporter |
| 11 | MG1655_b0832 | gsiD | gsiABCD glutathione ABC transporter |
| 11 | CFT073_phoH_c1159 | phoH | ATP-binding protein |
| 11 | MG1655_yciW_b1287 | yciW | predicted oxidoreductase |
| 11 | SAKAI_ECs2027 | ydcI | putative transcriptional regulator LysR-type |
| 11 | IG_1785137_1785468-r | NO_MATCH | Intergenic |
| 11 | MG1655_b1730 | ydjO | putative enzyme |
| 11 | MG1655_IS092_b4434 | isrB | small RNA |
| 11 | MG1655_fliY_b1920 | fliY | periplasmic cystine-binding protein; member of extracellular bacterial solute-binding protein family III |
| 11 | IG_2040048_2040359-r | NO_MATCH | Intergenic |
| 11 | MG1655_cbl_b1987 | cbl | Cbl transcriptional activator |
| 11 | IG_2234521_2234762-r | NO_MATCH | Intergenic |
| 11 | IG_2302414_2303127-r | NO_MATCH | Intergenic |
| 11 | MG1655_b2210 | mqo | malate:quinone oxidoreductase |
| 11 | IG_2304775_2304991-r | NO_MATCH | Intergenic |
| 11 | MG1655_b2354 | yfdK | hypothetical protein |
| 11 | MG1655_b2392 | mntH | MntH manganese ion NRAMP transporter |
| 11 | MG1655_cysK_b2414 | cysK | cysteine synthase |
| 11 | MG1655_cysM_b2421 | cysM | cysteine synthase B |
| 11 | IG_2651360_2651874-r | NO_MATCH | Intergenic |
| 11 | MG1655_lldP_b3603 | lldP | LldP lactate transporter |
| 11 | MG1655_lldR_b3604 | lldR | LldR transcriptional repressor |
| 11 | MG1655_lldD_b3605 | lldD | L-lactate:quinone oxidoreductase |
| 11 | MG1655_nlpA_b3661 | nlpA | lipoprotein-28 |
| 11 | MG1655_rarD_b3819 | rarD | predicted chloramphenicol resistance permease |
| 11 | MG1655_yigI_b3820 | yigI | conserved protein |
| 11 | MG1655_fadB_b3846 | fadB | dodecenoyl-CoA &delta;-isomerase, enoyl-CoA hydratase, 3-hydroxybutyryl-CoA epimerase, 3-hydroxyacyl-CoA dehydrogenase |
| 11 | MG1655_udhA_b3962 | sthA | pyridine nucleotide transhydrogenase, soluble |
|  |  |  |  |
| 12 | MG1655_yabJ_b0066 | thiQ | SfuC |
| 12 | MG1655_acnB_b0118 | acnB | aconitase B |
| 12 | MG1655_ykfB_b0250 | ykfB | hypothetical protein |
| 12 | IG_266192_266407-r | NO_MATCH | Intergenic |
| 12 | MG1655_prpD_b0334 | prpD | 2-methylcitrate dehydratase |
| 12 | IG_353817_354145-r | NO_MATCH | Intergenic |
| 12 | MG1655_mhpR_b0346 | mhpR | MhpR transcriptional regulator |
| 12 | IG_503921_504137-r | NO_MATCH | Intergenic |
| 12 | MG1655_b0484 | copA | YbaR |
| 12 | MG1655_ninE_b0548 | ninE | DLP12 prophage, conserved protein similar to phage 82 and lambda proteins |
| 12 | MG1655_fepE_b0587 | fepE | ferric enterobactin (enterochelin) transport |
| 12 | IG_637857_638167-r | NO_MATCH | Intergenic |
| 12 | IG_696357_696735-r | NO_MATCH | Intergenic |
| 12 | MG1655_nagB_b0678 | nagB | glucosamine-6-phosphate deaminase |
| 12 | MG1655_sdhA_b0723 | sdhA | succinate dehydrogenase flavoprotein |
| 12 | MG1655_sdhB_b0724 | sdhB | succinate dehydrogenase iron-sulfur protein |
| 12 | MG1655_b0725 | NO_MATCH | hypothetical protein |
| 12 | MG1655_sucA_b0726 | sucA | subunit of E1(0) component of 2-oxoglutarate dehydrogenase |
| 12 | MG1655_sucB_b0727 | sucB | SucB-lipoate |
| 12 | IG_761963_762236-f | NO_MATCH | Intergenic |
| 12 | MG1655_sucD_b0729 | sucD | succinyl-CoA synthetase, &alpha; subunit |
| 12 | MG1655_moaB_b0782 | moaB | MoaB subunit |
| 12 | IG_837149_837412-f | NO_MATCH | Intergenic |
| 12 | MG1655_glnQ_b0809 | glnQ | glutamine ABC transporter |
| 12 | MG1655_b0831 | gsiC | gsiABCD glutathione ABC transporter |
| 12 | IG_1050399_1050683-r | NO_MATCH | Intergenic |
| 12 | MG1655_rpmF_b1089 | rpmF | 50S ribosomal subunit protein L32 |
| 12 | IG_1160775_1161107-r | NO_MATCH | Intergenic |
| 12 | IG_1174389_1174649-r | NO_MATCH | Intergenic |
| 12 | MG1655_icdA_b1136 | icd | isocitrate dehydrogenase |
| 12 | IG_1269147_1269460-r | NO_MATCH | Intergenic |
| 12 | MG1655_ycjU_b1317 | pgmB | &beta;-phosphoglucomutase |
| 12 | MG1655_tehA_b1429 | tehA | TehA TDT transporter |
| 12 | MG1655_tehB_b1430 | tehB | tellurite resistance protein |
| 12 | IG_1521090_1521330-f | NO_MATCH | Intergenic |
| 12 | IG_1550016_1550421-f | NO_MATCH | Intergenic |
| 12 | MG1655_pqqL_b1494 | pqqL | putative zinc peptidase |
| 12 | IG_1580582_1580949-f | NO_MATCH | Intergenic |
| 12 | IG_1642368_1642674-f | NO_MATCH | Intergenic |
| 12 | MG1655_fumA_b1612 | fumA | fumarase A monomer |
| 12 | MG1655_b1630 | rsxD | integral membrane protein of SoxR-reducing complex |
| 12 | MG1655_b1631 | rsxG | member of SoxR-reducing complex |
| 12 | MG1655_nth_b1633 | nth | endonuclease III; specific for apurinic and/or apyrimidinic sites / endonulease |
| 12 | IG_1755135_1755444-f | NO_MATCH | Intergenic |
| 12 | MG1655_ynhG_b1678 | ynhG | putative ATP synthase subunit |
| 12 | MG1655_ppsA_b1702 | pps | PpsA |
| 12 | MG1655_yeaX_b1803 | yeaX | predicted oxidoreductase |
| 12 | CFT073_yedR_c2382 | yedR | predicted inner membrane protein |
| 12 | IG_2039141_2039396-r | NO_MATCH | Intergenic |
| 12 | IG_2244790_2245082-f | NO_MATCH | Intergenic |
| 12 | IG_2257317_2257738-f | NO_MATCH | Intergenic |
| 12 | SAKAI_ECs5484 | NO_MATCH | hypothetical protein |
| 12 | MG1655_b2225 | yfaP | conserved protein |
| 12 | MG1655_b2379 | yfdZ | predicted aminotransferase, PLP-dependent |
| 12 | IG_2516252_2516471-f | NO_MATCH | Intergenic |
| 12 | IG_2531401_2531783-r | NO_MATCH | Intergenic |
| 12 | MG1655_b2430 | yfeW | predicted periplasmic esterase |
| 12 | IG_2558919_2559387-r | NO_MATCH | Intergenic |
| 12 | MG1655_b2463 | maeB | malate dehydrogenase (oxaloacetate-decarboxylating) (NADP+) |
| 12 | MG1655_hmpA_b2552 | hmp | nitric oxide dioxygenase / dihydropteridine reductase |
| 12 | MG1655_kgtP_b2587 | kgtP | KgtP &alpha;-ketoglutarate MFS transporter |
| 12 | MG1655_b2655 | ygaR | predicted protein |
| 12 | MG1655_b2657 | yqaC | conserved protein |
| 12 | IG_2798497_2798743-f | NO_MATCH | Intergenic |
| 12 | MG1655_oraA_b2698 | recX | inhibitor of RecA |
| 12 | MG1655_b2756 | ygcH | predicted protein |
| 12 | IG_2885243_2885600-f | NO_MATCH | Intergenic |
| 12 | IG_2903441_2903663-f | NO_MATCH | Intergenic |
| 12 | MG1655_b2785 | rumA | 23S ribosomal RNA 5-methyluridine methyltransferase |
| 12 | MG1655_ygeH_b2852 | ygeH | predicted transcriptional regulator |
| 12 | IG_3010415_3010634-f | NO_MATCH | Intergenic |
| 12 | MG1655_b2878 | ygfK | putative oxidoreductase Fe-S subunit; possible component of selenate reductase / putative dihydrothymine dehydrogenase |
| 12 | MG1655_ygfQ_b2884 | ygfQ | predicted transporter |
| 12 | MG1655_ygiN_b3029 | ygiN | quinol monooxygenase monomer |
| 12 | CFT073_c3811 | NO_MATCH | hypothetical protein |
| 12 | MG1655_yqjI_b3071 | yqjI | predicted transcriptional regulator |
| 12 | MG1655_yraI_b3143 | yraI | putative chaperone |
| 12 | IG_3381904_3382337-r | NO_MATCH | Intergenic |
| 12 | CFT073_yhdL_c4052 | yhdL | conserved protein |
| 12 | MG1655_yhfV_b3379 | php | predicted hydrolase |
| 12 | MG1655_feoB_b3409 | feoB | FeoB ferrous iron transporter |
| 12 | MG1655_yhgG_b3410 | yhgG | conserved protein |
| 12 | MG1655_yhgI_b3414 | gntY | protein involved in utilization of DNA as a carbon source |
| 12 | IG_3663441_3663809-r | NO_MATCH | Intergenic |
| 12 | MG1655_yiaB_b3563 | yiaB | conserved inner membrane protein |
| 12 | MG1655_yicK_b3659 | setC | YicK MFS Transporter |
| 12 | MG1655_yidL_b3680 | yidL | putative ARAC-type regulatory protein |
| 12 | MG1655_fdoH_b3893 | fdoH | formate dehydrogenase-O, &beta; subunit |
| 12 | MG1655_fdoG_b3894 | fdoG | formate dehydrogenase-O, &alpha; subunit |
| 12 | MG1655_frwB_b3950 | frwB | EIIBCFrw |
| 12 | MG1655_yjbJ_b4045 | yjbJ | predicted stress response protein |
| 12 | CFT073_yjcC_c5052 | yjcC | conserved protein |
| 12 | MG1655_yjcF_b4066 | yjcF | conserved protein |
| 12 | MG1655_phnD_b4105 | phnD | alkylphosphonate ABC transporter |
| 12 | MG1655_ytfE_b4209 | ytfE | iron metabolism protein |
| 12 | MG1655_fbp_b4232 | fbp | fructose 1,6 bisphosphatase monomer |
| 12 | MG1655_fecE_b4287 | fecE | ferric dicitrate uptake system |
| 12 | MG1655_fecC_b4289 | fecC | ferric dicitrate uptake system |
| 12 | MG1655_fecB_b4290 | fecB | ferric dicitrate uptake system |
| 12 | MG1655_fimE_b4313 | fimE | regulator for fimA |
| 12 | MG1655_fimD_b4317 | fimD | outer membrane protein; export and assembly of type 1 fimbriae |
| 12 | MG1655_osmY_b4376 | osmY | hyperosmotically inducible periplasmic protein |
|  |  |  |  |
| 13 | MG1655_codB_b0336 | codB | CodB cytosine NCS1 transporter |
| 13 | MG1655_purK_b0522 | purK | N5-carboxyaminoimidazole ribonucleotide synthase monomer |
| 13 | MG1655_purE_b0523 | purE | N5-carboxyaminoimidazole ribonucleotide mutase |
| 13 | MG1655_add_b1623 | add | deoxyadenosine deaminase |
| 13 | MG1655_purT_b1849 | purT | GAR transformylase 2 |
| 13 | MG1655_purF_b2312 | purF | amidophosphoribosyl transferase |
| 13 | MG1655_cvpA_b2313 | cvpA | membrane protein required for colicin V production |
| 13 | MG1655_purC_b2476 | purC | phosphoribosylaminoimidazole-succinocarboxamide synthase |
| 13 | IG_2595639_2595850-r | NO_MATCH | Intergenic |
| 13 | MG1655_purM_b2499 | purM | phosphoribosylformylglycinamide cyclo-ligase |
| 13 | MG1655_purN_b2500 | purN | phosphoribosylglycinamide formyltransferase |
| 13 | MG1655_guaB_b2508 | guaB | IMP dehydrogenase |
| 13 | MG1655_purL_b2557 | purL | phosphoribosylformylglycinamide synthase |
| 13 | EDL933_purL_Z3835 | purL | phosphoribosylformylglycinamide synthase |
| 13 | MG1655_yfjW_b2642 | yfjW | CP4-57 prophage; predicted inner membrane protein |
| 13 | MG1655_gcvT_b2905 | gcvT | aminomethyltransferase |
| 13 | MG1655_yicE_b3654 | yicE | YicE NCS2 tranporter |
| 13 | MG1655_purD_b4005 | purD | phosphoribosylamine-glycine ligase |
| 13 | MG1655_purH_b4006 | purH | AICAR transformylase / IMP cyclohydrolase |
| 13 | MG1655_mcrC_b4345 | mcrC | MrcC subunit of 5-methylcytosine restriction system |
