# Supplementary material for: Rapid Changes in Gene Expression Dynamics in Response to Superoxide Reveal SoxRS-Dependent and Independent Transcriptional Networks
Source: PLoS One. 2007 Nov 14;2(11):e1186. doi: 10.1371/journal.pone.0001186 (PMC2064960; doi:10.1371/journal.pone.0001186)
Supplement: Supplemental Table S2 — Biological Relationship Analysis results for all 50 clusters. (0.26 MB DOC) [file pone.0001186.s004.doc]

Supplemental Table S2 – Biological Relationship Analysis results for all 50 clusters.

| Biological Relationship  Data Set | Cluster | e-score | Description |
| --- | --- | --- | --- |
| RegulonDB | 1 | 5.44E-08 | marA - DNA-binding transcriptional dual activator of multiple antibiotic resistance |
| RegulonDB | 1 | 2.69E-07 | rob - DNA-binding transcriptional activator |
| RegulonDB | 1 | 2.36E-09 | soxS - DNA-binding transcriptional dual regulator |
| RegulonDB | 2 | 1.53E-07 | marA - DNA-binding transcriptional dual activator of multiple antibiotic resistance |
| RegulonDB | 2 | 9.72E-04 | rob - DNA-binding transcriptional activator |
| RegulonDB | 2 | 5.97E-18 | soxS - DNA-binding transcriptional dual regulator |
| RegulonDB | 3 | 5.88E-05 | marA - DNA-binding transcriptional dual activator of multiple antibiotic resistance |
| RegulonDB | 3 | 1.06E-05 | soxS - DNA-binding transcriptional dual regulator |
| RegulonDB | 4 | 8.07E-04 | rpoD - RNA polymerase, sigma 70 (sigma D) factor |
| RegulonDB | 4 | 2.37E-22 | fur - DNA-binding transcriptional dual regulator of siderophore biosynthesis and transport |
| RegulonDB | 5 | 6.26E-06 | rpoD - RNA polymerase, sigma 70 (sigma D) factor |
| RegulonDB | 5 | 7.72E-03 | fecI - KpLE2 phage-like element; RNA polymerase, sigma 19 factor |
| RegulonDB | 5 | 2.65E-07 | oxyR - DNA-binding transcriptional dual regulator |
| RegulonDB | 5 | 2.76E-25 | fur - DNA-binding transcriptional dual regulator of siderophore biosynthesis and transport |
| RegulonDB | 6 | 1.43E-04 | oxyR - DNA-binding transcriptional dual regulator |
| RegulonDB | 6 | 4.75E-05 | birA - bifunctional biotin-[acetylCoA carboxylase] holoenzyme synthetase and DNA-binding transcriptional repressor, bio-5'-AMP-binding |
| RegulonDB | 6 | 1.14E-07 | iscR - DNA-binding transcriptional repressor |
| RegulonDB | 7 | 1.75E-21 | cysB - DNA-binding transcriptional dual regulator, O-acetyl-L-serine-binding |
| RegulonDB | 7 | 1.30E-03 | rpoD - RNA polymerase, sigma 70 (sigma D) factor |
| RegulonDB | 8 | 6.47E-06 | rpoH - RNA polymerase, sigma 32 (sigma H) factor |
| RegulonDB | 9 | 1.83E-04 | leuO - DNA-binding transcriptional activator |
| RegulonDB | 9 | 3.64E-03 | rpoD - RNA polymerase, sigma 70 (sigma D) factor |
| RegulonDB | 9 | 5.61E-03 | lrp - DNA-binding transcriptional dual regulator, leucine-binding |
| RegulonDB | 10 | 8.11E-03 | hdfR - DNA-binding transcriptional regulator |
| RegulonDB | 10 | 8.11E-03 | qseB - DNA-binding response regulator in two-component regulatory system with QseC |
| RegulonDB | 10 | 1.68E-03 | narL - DNA-binding response regulator in two-component regulatory system with NarX (or NarQ) |
| RegulonDB | 10 | 5.73E-07 | gatR - DNA-binding transcriptional regulator (pseudogene) |
| RegulonDB | 10 | 6.33E-06 | fnr - DNA-binding transcriptional dual regulator, global regulator of anaerobic growth |
| RegulonDB | 10 | 6.44E-08 | rpoD - RNA polymerase, sigma 70 (sigma D) factor |
| RegulonDB | 11 | 3.08E-06 | lldR - DNA-binding transcriptional repressor |
| RegulonDB | 11 | 2.36E-03 | cysB - DNA-binding transcriptional dual regulator, O-acetyl-L-serine-binding |
| RegulonDB | 11 | 3.99E-03 | arcA - DNA-binding response regulator in two-component regulatory system with ArcB or CpxA |
| RegulonDB | 11 | 1.69E-04 | pdhR - DNA-binding transcriptional dual regulator |
| RegulonDB | 12 | 3.14E-03 | fecI - KpLE2 phage-like element; RNA polymerase, sigma 19 factor |
| RegulonDB | 12 | 2.05E-03 | fnr - DNA-binding transcriptional dual regulator, global regulator of anaerobic growth |
| RegulonDB | 12 | 5.70E-04 | arcA - DNA-binding response regulator in two-component regulatory system with ArcB or CpxA |
| RegulonDB | 13 | 4.83E-05 | rpoD - RNA polymerase, sigma 70 (sigma D) factor |
| RegulonDB | 13 | 1.03E-25 | purR - DNA-binding transcriptional repressor, hypoxanthine-binding |
| RegulonDB | 19 | 1.63E-03 | betI - DNA-binding transcriptional repressor |
| RegulonDB | 28 | 1.32E-03 | fis - global DNA-binding transcriptional dual regulator |
| RegulonDB | 31 | 5.29E-06 | fis - global DNA-binding transcriptional dual regulator |
| RegulonDB | 39 | 1.43E-05 | fnr - DNA-binding transcriptional dual regulator, global regulator of anaerobic growth |
| RegulonDB | 39 | 9.27E-03 | arcA - DNA-binding response regulator in two-component regulatory system with ArcB or CpxA |
| RegulonDB | 49 | 4.23E-03 | rpoE - RNA polymerase, sigma 24 (sigma E) factor |
| ASAP_GO | 3 | 1.71E-05 | GO:0042493 response to drug |
| ASAP_GO | 4 | 5.71E-09 | GO:0009239 enterobactin biosynthesis |
| ASAP_GO | 4 | 4.00E-08 | GO:0006826 iron ion transport |
| ASAP_GO | 4 | 1.37E-07 | GO:0019184 nonribosomal peptide biosynthesis |
| ASAP_GO | 5 | 4.15E-03 | GO:0009239 enterobactin biosynthesis |
| ASAP_GO | 5 | 7.21E-10 | GO:0006826 iron ion transport |
| ASAP_GO | 6 | 2.67E-05 | GO:0009102 biotin biosynthesis |
| ASAP_GO | 6 | 4.55E-05 | GO:0006826 iron ion transport |
| ASAP_GO | 7 | 3.11E-21 | GO:0006790 sulfur metabolism |
| ASAP_GO | 8 | 5.94E-03 | GO:0006457 protein folding |
| ASAP_GO | 9 | 8.21E-04 | GO:0009088 threonine biosynthesis |
| ASAP_GO | 9 | 2.13E-04 | GO:0009098 leucine biosynthesis |
| ASAP_GO | 10 | 1.99E-05 | GO:0042330 taxis |
| ASAP_GO | 10 | 1.24E-05 | GO:0009296 flagella biogenesis |
| ASAP_GO | 10 | 5.41E-04 | GO:0016052 carbohydrate catabolism |
| ASAP_GO | 11 | 2.56E-07 | GO:0019344 cysteine biosynthesis |
| ASAP_GO | 12 | 6.18E-09 | GO:0006099 tricarboxylic acid cycle |
| ASAP_GO | 12 | 8.68E-04 | GO:0006826 iron ion transport |
| ASAP_GO | 13 | 2.48E-25 | GO:0006164 purine nucleotide biosynthesis |
| ASAP_GO | 13 | 4.51E-04 | GO:0015949 nucleobase, nucleoside and nucleotide interconversion |
| ASAP_GO | 27 | 4.00E-03 | GO:0006308 DNA catabolism |
| ASAP_GO | 31 | 6.28E-27 | GO:0006412 protein biosynthesis |
| ASAP_GO | 36 | 6.85E-09 | GO:0042330 taxis |
| ASAP_GO | 36 | 6.72E-11 | GO:0009296 flagella biogenesis |
| ASAP_GO | 39 | 1.79E-03 | GO:0009060 aerobic respiration |
| Eco_Cyc | 3 | 4.13E-03 | YhbG/YhbN ABC transporter |
| Eco_Cyc | 3 | 5.19E-04 | arginine ABC transporter |
| Eco_Cyc | 3 | 5.28E-05 | AcrAB-MDR-CPLX |
| Eco_Cyc | 4 | 5.71E-09 | enterobactin biosynthesis |
| Eco_Cyc | 4 | 5.47E-07 | enterobactin synthase multienzyme complex |
| Eco_Cyc | 5 | 8.45E-04 | ribonucleoside-diphosphate reductase II |
| Eco_Cyc | 5 | 4.34E-09 | ferrichrome uptake system |
| Eco_Cyc | 5 | 3.45E-08 | ferric enterobactin ABC transporter |
| Eco_Cyc | 5 | 5.03E-03 | Outer Membrane Ferrichrome Transport System |
| Eco_Cyc | 5 | 1.15E-08 | Ferric Enterobactin Transport System |
| Eco_Cyc | 5 | 5.50E-06 | SufB-SufC-SufD cysteine desulfurase (SufS) activator complex |
| Eco_Cyc | 5 | 5.50E-06 | iron (III) hydroxamate ABC transporter |
| Eco_Cyc | 6 | 3.96E-03 | TonB energy transducing system |
| Eco_Cyc | 6 | 7.86E-03 | vitamin B12 outer membrane transport complex |
| Eco_Cyc | 6 | 5.15E-05 | Outer Membrane Ferric Citrate Transport System |
| Eco_Cyc | 6 | 7.86E-03 | Outer Membrane Ferrichrome Transport System |
| Eco_Cyc | 6 | 7.86E-03 | Outer Membrane Ferric Enterobactin Transport System |
| Eco_Cyc | 6 | 5.15E-05 | biotin biosynthesis I |
| Eco_Cyc | 6 | 7.00E-04 | ferric dicitrate uptake system |
| Eco_Cyc | 7 | 5.45E-15 | sulfate assimilation |
| Eco_Cyc | 7 | 7.77E-05 | sulfate adenylyltransferase |
| Eco_Cyc | 7 | 7.77E-05 | flavin reductase / sulfite reductase-(NADPH) |
| Eco_Cyc | 7 | 7.64E-10 | sulfate ABC transporter |
| Eco_Cyc | 7 | 7.64E-10 | thiosulfate ABC transporter |
| Eco_Cyc | 8 | 7.69E-03 | HslVU protease |
| Eco_Cyc | 9 | 2.02E-04 | leucine biosynthesis |
| Eco_Cyc | 9 | 1.20E-03 | BasSR Two-Component Signal Transduction System |
| Eco_Cyc | 9 | 1.20E-03 | threonine biosynthesis from homoserine |
| Eco_Cyc | 9 | 7.12E-03 | phosphate ABC transporter |
| Eco_Cyc | 10 | 8.85E-03 | tagatose-1,6-bisphosphate aldolase 2 |
| Eco_Cyc | 10 | 8.85E-03 | cytochrome <i>bd</i>-I terminal oxidase |
| Eco_Cyc | 10 | 2.62E-03 | galactitol degradation |
| Eco_Cyc | 11 | 8.77E-06 | YliA/YliB/YliC/YliD ABC transporter |
| Eco_Cyc | 12 | 3.90E-04 | iron dicitrate ABC transporter |
| Eco_Cyc | 12 | 3.47E-09 | TCA cycle -- aerobic respiration |
| Eco_Cyc | 12 | 6.07E-03 | catalytic subcomplex of succinate dehydrogenase |
| Eco_Cyc | 12 | 5.21E-03 | ferric dicitrate uptake system |
| Eco_Cyc | 13 | 6.64E-22 | purine nucleotides _de novo_ biosynthesis I |
| Eco_Cyc | 17 | 1.50E-03 | AtoSC Two-Component Signal Transduction System |
| Eco_Cyc | 31 | 5.62E-03 | Sec Protein Secretion Complex |
| Eco_Cyc | 31 | 9.30E-04 | SecD-SecF-Yajc-YidC Secretion Complex |
| Eco_Cyc | 35 | 7.91E-04 | GspC-O secretoncomplex |
| Eco_Cyc | 39 | 4.98E-04 | NADH dehydrogenase I |
| Eco_Cyc | 41 | 8.08E-03 | membrane-bound subcomplex of succinate dehydrogenase |
| KEGG | 4 | 3.99E-08 | Biosynthesis of siderophore group nonribosomal peptides [PATH:ko01053] |
| KEGG | 4 | 8.05E-07 | Biosynthesis of Polyketides and Nonribosomal Peptides |
| KEGG | 4 | 5.00E-05 | Pores ion channels [TC:1] |
| KEGG | 5 | 6.77E-03 | ABC transporters [PATH:ko02010] [BR:ko02000] [TC:3.A.1] |
| KEGG | 6 | 2.46E-04 | Biotin metabolism [PATH:ko00780] |
| KEGG | 7 | 6.96E-03 | ABC transporters [PATH:ko02010] [BR:ko02000] [TC:3.A.1] |
| KEGG | 7 | 1.69E-11 | Sulfur metabolism [PATH:ko00920] |
| KEGG | 7 | 1.67E-08 | Selenoamino acid metabolism [PATH:ko00450] |
| KEGG | 7 | 1.34E-05 | Metabolism of Other Amino Acids |
| KEGG | 7 | 1.85E-04 | Energy Metabolism |
| KEGG | 8 | 3.20E-03 | Folding, Sorting and Degradation |
| KEGG | 8 | 4.55E-05 | Protein folding and associated processing |
| KEGG | 9 | 7.01E-04 | Amino Acid Metabolism |
| KEGG | 10 | 4.03E-07 | Bacterial motility proteins [BR:ko02035] |
| KEGG | 10 | 3.08E-05 | Cellular Processes |
| KEGG | 10 | 6.05E-03 | Valine, leucine and isoleucine biosynthesis [PATH:ko00290] |
| KEGG | 10 | 4.03E-07 | Cell Motility |
| KEGG | 10 | 3.26E-08 | Flagellar assembly [PATH:ko02040] |
| KEGG | 12 | 3.12E-07 | Citrate cycle (TCA cycle) [PATH:ko00020] [GO:0006099] |
| KEGG | 12 | 4.53E-06 | Reductive carboxylate cycle (CO2 fixation) [PATH:ko00720] |
| KEGG | 12 | 1.29E-04 | Carbohydrate Metabolism |
| KEGG | 12 | 3.28E-03 | Energy Metabolism |
| KEGG | 13 | 1.01E-15 | Purine metabolism [PATH:ko00230] |
| KEGG | 13 | 3.12E-05 | Metabolism |
| KEGG | 13 | 1.58E-13 | Nucleotide Metabolism |
| KEGG | 28 | 5.69E-03 | Translation |
| KEGG | 28 | 4.06E-04 | Ribosome [PATH:ko03010] [BR:ko03010] |
| KEGG | 31 | 7.09E-35 | Translation |
| KEGG | 31 | 3.75E-40 | Ribosome [PATH:ko03010] [BR:ko03010] |
| KEGG | 31 | 1.68E-19 | Genetic Information Processing |
| KEGG | 36 | 1.86E-09 | Bacterial motility proteins [BR:ko02035] |
| KEGG | 36 | 2.62E-07 | Cellular Processes |
| KEGG | 36 | 1.86E-09 | Cell Motility |
| KEGG | 36 | 9.68E-03 | Type III secretion system [PATH:ko03070] |
| KEGG | 36 | 1.07E-10 | Flagellar assembly [PATH:ko02040] |
| KEGG | 39 | 3.93E-04 | Oxidative phosphorylation [PATH:ko00190] |
| KEGG | 40 | 6.26E-03 | Transcription |
| KEGG | 40 | 5.48E-03 | Type II diabetes mellitus [PATH:ko04930] |
| KEGG | 40 | 4.55E-03 | Amino Acid Metabolism |
| KEGG | 47 | 1.50E-07 | Metabolism |
| KEGG | 47 | 2.35E-07 | Amino Acid Metabolism |
| KEGG | 47 | 1.23E-03 | Methionine metabolism [PATH:ko00271] |
| KEGG | 49 | 5.97E-04 | Metabolism |
| KEGG | 49 | 8.30E-03 | Lipid Metabolism |
| Operon | 1 | 9.03E-05 | b2253 b2254 b2255 b2256 b2257 b2258 |
| Operon | 2 | 5.16E-05 | ybjC mdaA rimK ybjN |
| Operon | 2 | 4.07E-03 | mutY yggX mltC |
| Operon | 2 | 1.36E-03 | pqiA pqiB |
| Operon | 2 | 1.30E-05 | ybaO mdlA mdlB |
| Operon | 3 | 5.83E-04 | artP artI artQ artM artJ |
| Operon | 3 | 7.46E-07 | ycgZ ymgA ymgB ymgC |
| Operon | 3 | 1.10E-05 | yrbG yrbH yrbI yrbK yhbN yhbG |
| Operon | 3 | 4.65E-03 | b2680 b2681 |
| Operon | 3 | 4.65E-03 | acrA acrB |
| Operon | 4 | 2.04E-04 | fes entF fepE |
| Operon | 4 | 6.80E-05 | b0805 ybiX |
| Operon | 4 | 4.06E-04 | nrdH nrdI nrdE nrdF |
| Operon | 4 | 2.05E-06 | entC entE entB entA ybdB |
| Operon | 5 | 2.17E-09 | ydiC ynhE ynhD ynhC b1680 ynhA ynhG |
| Operon | 5 | 1.73E-08 | fhuA fhuC fhuD fhuB |
| Operon | 5 | 4.23E-04 | fecI fecR |
| Operon | 5 | 4.23E-04 | b1016 b1017 ycdO ycdB |
| Operon | 5 | 2.51E-03 | nrdH nrdI nrdE nrdF |
| Operon | 5 | 2.75E-06 | fepD fepG fepC |
| Operon | 5 | 4.17E-03 | entC entE entB entA ybdB |
| Operon | 6 | 1.79E-05 | yfhJ pepB sseB |
| Operon | 6 | 1.79E-05 | yfhE hscA fdx |
| Operon | 6 | 1.84E-03 | b3007 exbB exbD |
| Operon | 6 | 8.49E-07 | b2532 b2531 yfhO b2529 yfhF |
| Operon | 6 | 1.84E-03 | hscA fdx |
| Operon | 6 | 1.84E-03 | yhaO yhaN |
| Operon | 6 | 5.48E-03 | bioB bioF bioC bioD |
| Operon | 7 | 1.69E-07 | cysJ cysI cysH |
| Operon | 7 | 1.69E-07 | cysD cysN cysC |
| Operon | 7 | 5.18E-05 | yeeE yeeD |
| Operon | 7 | 2.54E-09 | cysP cysU cysW cysA cysM |
| Operon | 9 | 2.86E-03 | b2611 ypjE yfjD |
| Operon | 9 | 9.43E-03 | pstS pstC pstA pstB phoU |
| Operon | 9 | 3.28E-05 | thrL thrA thrB thrC |
| Operon | 9 | 5.69E-03 | yjdE yjdB basR basS |
| Operon | 9 | 8.15E-05 | leuL leuA leuB leuC leuD |
| Operon | 10 | 2.98E-05 | ycbR ycbS b0941 b0942 b0943 ycbF |
| Operon | 10 | 2.41E-03 | hemA prfA hemK b1213 ychA kdsA |
| Operon | 10 | 1.24E-04 | fruB fruK fruA |
| Operon | 10 | 8.11E-03 | flhD flhC |
| Operon | 10 | 8.11E-03 | cydA cydB |
| Operon | 10 | 1.66E-07 | gatY gatZ gatA gatB gatC gatD gatR2 |
| Operon | 10 | 2.41E-03 | hemA prfA hemK b1213 ychA kdsA |
| Operon | 11 | 1.80E-03 | yigI rarD yigG yigF |
| Operon | 11 | 4.89E-08 | ybiK b0829 b0830 b0831 b0832 |
| Operon | 11 | 1.76E-06 | lldP lldR lldD |
| Operon | 12 | 5.44E-08 | sdhC sdhD sdhA sdhB b0725 sucA sucB sucC sucD |
| Operon | 12 | 1.66E-03 | fecA fecB fecC fecD fecE |
| Operon | 13 | 1.32E-04 | cvpA purF |
| Operon | 13 | 1.32E-04 | cvpA purF |
| Operon | 13 | 1.32E-04 | purE purK |
| Operon | 13 | 1.32E-04 | purH purD |
| Operon | 13 | 1.32E-04 | purM purN |
| Operon | 17 | 3.42E-03 | atoS atoC |
| Operon | 23 | 4.50E-03 | acs yjcH yjcG |
| Operon | 24 | 6.29E-03 | rnc era recO pdxJ acpS |
| Operon | 25 | 4.16E-03 | rtcB rtcA yhgJ |
| Operon | 31 | 7.27E-10 | rpsJ rplC rplD rplW rplB rpsS rplV rpsC rplP rpmC rpsQ |
| Operon | 31 | 8.68E-03 | rpsM rpsK rpsD rpoA rplQ |
| Operon | 31 | 2.28E-13 | rplN rplX rplE rpsN rpsH rplF rplR rpsE rpmD rplO prlA rpmJ |
| Operon | 31 | 1.82E-03 | rpsF priB rpsR rplI |
| Operon | 32 | 6.66E-03 | b2611 ypjE yfjD |
| Operon | 35 | 2.60E-03 | yheE yheF yheG hofF hofG hofH yheH yheI yheJ yheK pshM hofD |
| Operon | 36 | 1.31E-03 | fliF fliG fliH fliI fliJ fliK |
| Operon | 36 | 5.56E-03 | yohJ yohK |
| Operon | 36 | 2.23E-06 | flgB flgC flgD flgE flgF flgG flgH flgI flgJ flgK |
| Operon | 39 | 7.85E-04 | nuoA nuoB nuoC nuoE nuoF nuoG nuoH nuoI nuoJ nuoK nuoL nuoM nuoN |
| Operon | 44 | 3.04E-03 | yabP yabQ |
| Operon | 47 | 1.77E-03 | yfcB aroC mepA yfcA b2326 b2325 |
