# Supplementary material for: Rapid Changes in Gene Expression Dynamics in Response to Superoxide Reveal SoxRS-Dependent and Independent Transcriptional Networks
Source: PLoS One. 2007 Nov 14;2(11):e1186. doi: 10.1371/journal.pone.0001186 (PMC2064960; doi:10.1371/journal.pone.0001186)
Supplement: Supplemental Table S3 — Human homologs of SoxS regulated genes identified by BlastP. (0.06 MB DOC) [file pone.0001186.s005.doc]

Supplemental Table S3. Human homologs of SoxS regulated genes identified by BlastP.

| Gene | *E. coli* Description | e-value | Human Description |
| --- | --- | --- | --- |
| *acnA* | aconitase | 0 | aconitase 1 |
| *pgi* | phosphoglucose isomerase | 0 | glucose phosphate isomerase |
| *fumC* | fumarase C monomer | 1.00E-141 | fumarate hydratase precursor |
| *feaB* | putative succinate-semialdehyde dehydrogenase | 1.00E-90 | aldehyde dehydrogenase 1A1 |
| *zwf* | glucose 6-phosphate-1-dehydrogenase | 4.00E-83 | glucose-6-phosphate dehydrogenase |
| *treF* | trehalase, cytoplasmic | 6.00E-74 | trehalase (brush-border membrane glycoprotein) |
| *mdl* | MdlB | 1.00E-72 | ATP-binding cassette, sub-family B (MDR/TAP), member 9 isoform 1 |
| *mdlA* | MdlA | 8.00E-64 | ATP-binding cassette, sub-family B (MDR/TAP), member 9 isoform 1 |
| *map* | methionine aminopeptidase | 7.00E-63 | methionyl aminopeptidase 1 |
| *hemB* | porphobilinogen synthase | 1.00E-56 | delta-aminolevulinic acid dehydratase isoform b |
| *sodA* | superoxide dismutase (Mn) | 4.00E-44 | manganese superoxide dismutase isoform A precursor |
| *astD* | succinylglutamic semialdehyde dehydrogenase | 1.00E-39 | aldehyde dehydrogenase 5A1 precursor, isoform 2 |
| *yagR* | putative molybdemum cofactor-binding oxidoreductase | 1.00E-30 | xanthine dehydrogenase |
| *dgoT* | YidT galactonate MFS transporter | 4.00E-25 | solute carrier family 17 (anion/sugar transporter), member 5 |
| *poxB* | pyruvate oxidase monomer | 3.00E-24 | 2-hydroxyphytanoyl-CoA lyase |
| *yhbG* | YhbG/YhbN ABC transporter | 3.00E-24 | ATP-binding cassette, sub-family A member 4 |
| *rimK* | ribosomal protein S6 modification protein | 2.00E-21 | family with sequence similarity 80, member A |
| *artP* | arginine ABC transporter | 9.00E-19 | ATP-binding cassette, sub-family B, member 7 |
| *ybaL* | YbaL CPA2 transporter | 2.00E-17 | transmembrane and coiled-coil domains 3 |
| *yadR* | conserved hypothetical protein | 4.00E-16 | HESB like domain containing 1 |
| *kdsC* | 3-deoxy-D-manno-octulosonate 8-phosphate phosphatase | 8.00E-16 | cytidine 5'-monophosphate N-acetylneuraminic acid synthetase |
| *chaC* | cation transport regulator | 2.00E-14 | hypothetical protein LOC79094 |
| *nhoA* | N-hydroxyarylamine O-acetyltransferase | 1.00E-11 | similar to Arylamine N-acetyltransferase |
| *yhjX* | YhjX MFS transporter | 3.00E-10 | monocarboxylate transporter 13 |
| *yeiI* | putative kinase | 3.00E-09 | ribokinase |
| *idi* | isopentenyl diphosphate isomerase | 3.00E-09 | isopentenyl-diphosphate delta isomerase |
| *ligA* | DNA ligase | 3.00E-08 | replication factor C large subunit |
| *fpr* | flavodoxin NADP+ reductase | 4.00E-06 | cytochrome b5 reductase isoform 1 |
| *cmr* | MdfA/Cmr MFS multidrug transporter | 4.00E-05 | solute carrier family 18 (vesicular acetylcholine), member 3 |
| *mdtG* | YceE drug MFS transporter | 9.00E-05 | hypothetical protein LOC84804 |
| *yicM* | YicM MFS transporter | 2.00E-04 | solute carrier family 16, member 7 |
| *ychA* | hypothetical protein | 4.00E-04 | F-box only protein 21 isoform 1 |
| *ygfZ* | conserved protein | 4.00E-04 | hypothetical protein LOC200205 |
| *pncB* | nicotinate phosphoribosyltransferase | 7.00E-04 | nicotinate phosphoribosyltransferase |
