# Supplementary figures and images for: Correction: Rapid Changes in Gene Expression Dynamics in Response to Superoxide Reveal SoxRS-Dependent and Independent Transcriptional Networks
Source: PLoS One. 2012 Nov 8;7(11):10.1371/annotation/5cba04eb-5172-43a7-ad92-10efcd3858c9. doi: 10.1371/annotation/5cba04eb-5172-43a7-ad92-10efcd3858c9 (PMC3525699; doi:10.1371/annotation/5cba04eb-5172-43a7-ad92-10efcd3858c9)

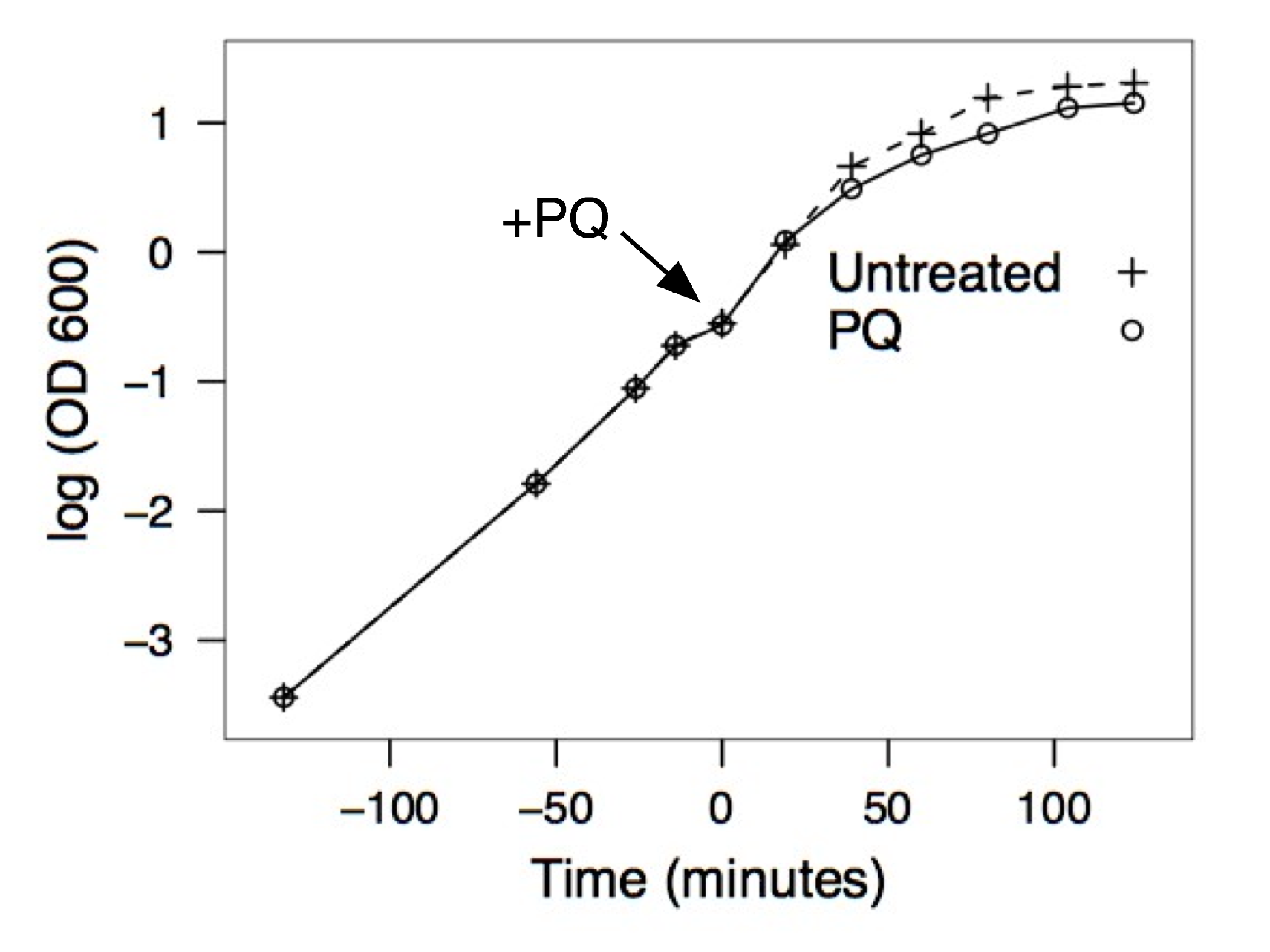

Supplement: Supplementary file 1 [file pone.5cba04eb-5172-43a7-ad92-10efcd3858c9.s001.tif]
